# Electrolyte Chemistry Development for Sodium‐Based Batteries: A Blueprint from Lithium or a Step Toward Originality?

**DOI:** 10.1002/anie.202424543

**Published:** 2025-04-14

**Authors:** Ziyu Song, Zhirong Xing, Jiaxun Yang, Jiayi Chen, Weican Hu, Pu Li, Wenfang Feng, Gebrekidan Gebresilassie Eshetu, Egbert Figgemeier, Stefano Passerini, Michel Armand, Zhibin Zhou, Heng Zhang

**Affiliations:** ^1^ Key Laboratory of Material Chemistry for Energy Conversion and Storage (Ministry of Education) School of Chemistry and Chemical Engineering Huazhong University of Science and Technology Wuhan 430074 China; ^2^ Institute of Power Electronics and Electric Drives (ISEA) Center for Ageing Reliability and Lifetime Prediction of Electrochemical and Power Electronic Systems (CARL) RWTH Aachen University Campus Boulevard 89 52074 Aachen Germany; ^3^ Department of Material Science and Engineering, Mekelle Institute of Technology Mekelle University P.O. Box 1632 Mekelle Ethiopia; ^4^ Helmholtz‐Institute Münster (HI MS) IMD‐4 Forschungszentrum Jülich, 46 48149 Münster Germany; ^5^ Austrian Institute of Technology (AIT) Center for Transport Technologies Giefinggasse 4 Wien 1020 Austria; ^6^ Centre for Cooperative Research on Alternative Energies (CIC energiGUNE) Basque Research and Technology Alliance (BRTA) Vitoria‐Gasteiz 01510 Spain

**Keywords:** Aluminum corrosion, Chemical stability, Electrolyte, Rechargeable sodium‐based battery, Solvation

## Abstract

Currently, electrolyte design for sodium‐based batteries is largely inherited from their lithium‐based counterparts, which often present critical challenges that hinder forging new perspectives and thus further improvements. This work delves into the key properties of representative sodium‐ and lithium‐based electrolytes, encompassing prevailing salt anions. It aims to evaluate the impact of cation chemistry, including their nature and the degree of interactions with counter anions, thereby bridging the gap in effectively transferring the know‐how accumulated in lithium batteries to sodium‐based batteries. The results demonstrate that the unique impact of salt anions on the properties of metal‐ion conducting electrolytes is tightly correlated with the nature of metal cations. By synchronizing the anionic structures with the critical features of sodium cations, the solvating dynamics and transport properties, chemical stability, aluminum corrosion behavior, and other key properties of the electrolytes could be finely tuned to fit the specific requirements of advanced sodium‐based batteries. This work gives an in‐depth insight into the chemical and physical features of sodium‐based electrolytes, with a potential avenue to accelerate the deployment of high‐performance sodium batteries and simultaneously inspire and guide the design of other electrolytes for emerging mono‐ and multivalent cation‐based rechargeable batteries.

## Graphical abstract

Unique solvation environment of sodium cations, brought by their larger ion volume and lower charge density, significantly impacts the physicochemical and interfacial properties of sodium‐based electrolytes, highlighting the crucial importance of moving beyond mere mimicry of lithium‐based designs.

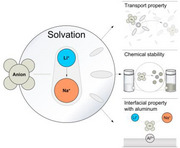

## Introduction

In the search for sustainable and affordable energy storage devices, room‐temperature rechargeable sodium batteries (RSBs) have emerged as enthralling alternatives to state‐of‐the‐art lithium‐ion batteries (LIBs).^[^ ^1^, ^2^, ^3^ ^]^ Since sodium is positioned directly below lithium in Group IA of the periodic table, it naturally exhibits similar physical and chemical properties to lithium. However, unlike lithium, sodium is abundant (∼23 000 ppm (sodium) vs. ∼17 ppm (lithium) in the earth's crust),^[^ ^4^, ^5^, ^6^ ^]^ widely available, and evenly distributed all over the world (including in seawater, salt lakes, minerals, etc.).^[^ ^7^, ^8^, ^9^ ^]^ This conceivably relieves disquiets related to resource scarcity and geopolitical dependence.^[^ ^4^, ^5^, ^6^ ^]^ Another factor is that cobalt and nickel are not required for many sodium‐based batteries. Instead, more abundant iron‐ and manganese‐based cathode materials are used.^[^ ^10^ ^]^ Even more delectable is the possible use of the copiously available, cheaper, and lighter aluminum (Al), in place of copper, as a negative current collector,^[^ ^11^, ^12^ ^]^ and hard carbon from renewable sources in place of graphite for the negative electrode.^[^ ^13^ ^]^ RSBs have several advantages over LIBs, including environmental abundance, low cost of precursors, higher rate capability, better temperature adaptability, etc.^[^ ^14^, ^15^, ^16^, ^17^, ^18^, ^19^ ^]^ Moreover, with some modifications, RSBs can adopt existing production lines and infrastructure of LIB technology, and thus with no additional capital or even less (equipment and process) investment.^[^ ^7^, ^8^, ^9^ ^]^

**Figure 1:**
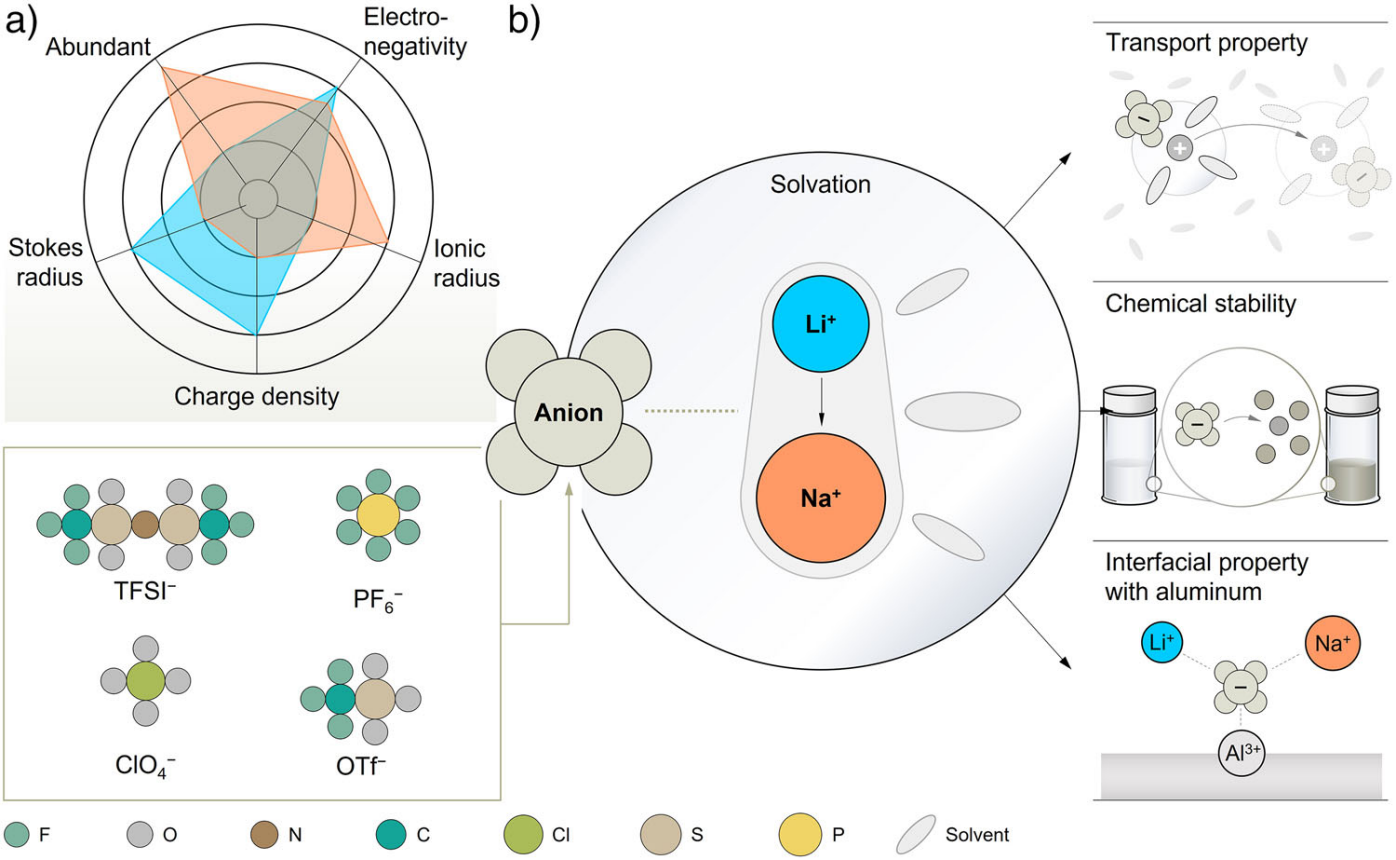
Schematic diagram for the electrolyte chemistry associated with rechargeable sodium and lithium batteries. a) Unique features of sodium battery technology as compared to its lithium precursor. b) Possible impacts brought by replacing lithium cations in the prevailing lithium‐based electrolyte with their sodium successor. The abbreviations are given below: bis(trifluoromethanesulfonyl)imide anion [(CF_3_SO_2_)_2_N^−^, TFSI^−^), hexafluorophosphate anion (PF_6_^−^), perchlorate anion (ClO_4_^−^), and trifluoromethanesulfonate anion (CF_3_SO_3_^−^, OTf^−^).

Though both lithium and sodium belong to the same IA group in the periodic table, RSBs are characterized by several fundamental differences from LIBs, bestowing them both advantages and disadvantages.^[^ ^20^, ^21^, ^22^ ^]^ These fundamental differences include larger ionic radius (Shannon ionic radius: 102 pm (Na^+^) vs. 76 pm (Li^+^)),^[^ ^23^ ^]^ lower charge density (36 C mm^−3^ (Na^+^) vs. 87 C mm^−3^ (Li^+^)),^[^ ^24^ ^]^ higher redox potential (e.g., in propylene carbonate, −2.6 V (Na/Na^+^) vs. −2.8 V (Li/Li^+^) vs. standard hydrogen electrode),^[^ ^25^ ^]^ etc. (Figure 1a).^[^ ^26^, ^27^ ^]^ These peculiar characteristics severely affect key atomic and material‐level properties and thus cell‐level performance metrics, including safety, cycle life, energy density, and rate capability.^[^ ^28^ ^]^ For instance, due to the size mismatch between Na^+^ cation and the graphite interlayer spacings, state‐of‐the‐art graphite, which is the standard electrode material for LIBs, cannot be used for RSBs.^[^ ^29^, ^30^ ^]^

It is noteworthy to mention that electrolytes are ubiquitous and indispensable components of electrochemical devices that dictate the bulk and interfacial properties and electrochemistry, and thus the overall performance of batteries, including practically obtainable capacity, rate capability, thermal stress, and lifetime.^[^ ^31^, ^32^ ^]^ Yet, most of the research work in RSBs has been dedicated to investigating electrode materials, following the same metamorphic trend of LIBs.^[^ ^33^ ^]^ Most importantly, while the lessons learned in the last four decades on LIBs, including their electrolytes, could help to mimic and understand components of RSBs, there is no guarantee of a direct transfer of knowledge and experience from lithium to sodium battery chemistry. This also means that electrolyte constituents (solvents, salt anions, and additives) that perform well for lithium batteries may not be suitable for sodium or vice versa. Moreover, the differences between lithium and sodium electrolytes, especially the decisive electrolyte components, the salt anions, and their interactions with cations, have not yet been thoroughly explored and validated. Thus, investigating and fostering an in‐depth understanding of key indicators of electrolyte properties such as the ion solvation, ion transport, charge transfer, thermal‐induced chemical stability and thus safety, cost, phase change behavior, sacrificial reactions of sodium electrolytes in the vicinity of the active materials and current collector, and benchmarking them with their lithium counterparts is of supreme importance to accelerate the large‐scale deployment of RSBs.^[^ ^34^ ^]^

To discern the impact of the nature of cations and their corresponding interactions with the anions, thus establishing solid foundation to bridge the gap for efficiently transferring the knowledge accumulated in lithium batteries to their successors (e.g., sodium‐based systems), the current work delves into key properties of representative sodium and lithium electrolytes based on prevailing salt anions (Figure 1b), enlisting bis(trifluoromethanesulfonyl)imide (TFSI^−^), hexafluorophosphate (PF_6_^−^), perchlorate (ClO_4_^−^), and trifluoromethanesulfonate (OTf^−^). Ethylene carbonate (EC)/ethyl methyl carbonate (EMC; 3:7, v/v) mixture, a standard solvation medium for both LIBs and RSBs, is selected for this work.^[^ ^32^ ^]^ In addition, a fixed salt concentration of 0.5 M is adopted to ensure the full dissolution of conducting salts as the solubility of sodium‐based salts is slightly lower than their lithium counterparts. The physical (e.g., viscosity, ionic conductivity, and solvation dynamics), chemical, and electrochemical properties (e.g., chemical stability and aluminum corrosion behavior) of the sodium and lithium electrolytes are systematically evaluated, characterized, and juxtaposed. The study carefully inspects the similarities and differences between sodium and lithium electrolytes, providing not only a suitable reference standard for the evaluation of sodium‐based electrolytes but also a guiding rule toward the screening and development of new functional electrolytes for advanced sodium and other emerging mono‐ (e.g., potassium) and multivalent (e.g., calcium, magnesium, aluminum, etc.) cation‐based electrolytes.

## Results and Discussion

### Ion Transport and Solvation Dynamics

**Figure 2:**
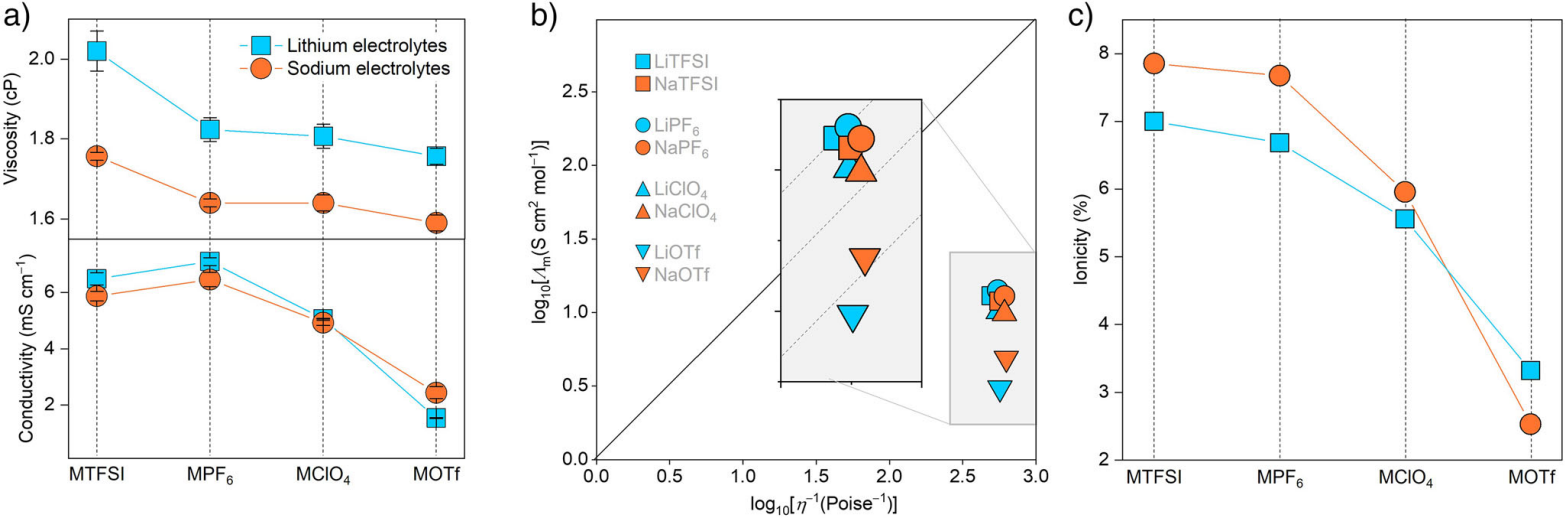
Transport behavior of the sodium and lithium electrolytes. a) Viscosity and ionic conductivity obtained at 25 °C for the as‐prepared electrolytes. The error bars represent experimental errors from three independent replicates. b) Walden plot for the two families of electrolytes, in which the molar ionic conductivity (*Λ*_m_) and viscosity (*η*) values shown herein are measured at 25 °C. c) Comparison of ion association in lithium and sodium electrolytes.

Foremost, the ion transport behaviors of the as‐prepared sodium and lithium‐based electrolytes involving different salt anions are evaluated, and the phase transition properties are presented in Supporting Information (see Figure S1 and Table S1; cf. Supporting Discussion #1). Figure 2a displays the viscosity (*η*) of the as‐prepared electrolytes measured at 25 °C. For lithium‐based samples, the viscosity decreases in the order of LiTFSI (2.02 ± 0.05 cP) > LiPF_6_ (1.82 ± 0.03 cP) > LiClO_4_ (1.79 ± 0.03 cP) > LiOTf (1.76 ± 0.02 cP). In general, the viscosity of an electrolyte is a macroscopic manifestation of the microscopic interactions between the electrolyte components (i.e., solvents, solvent–cation, solvent–anion, solvated ion pair, etc.) and is primarily related to factors enlisting the proportion of the components, ion association, and the properties of the solvates.^[^ ^35^, ^36^ ^]^ By changing salt anion, a noticeable variation of viscosity values is observed for both lithium and sodium‐based electrolytes (Figure 2a), which indicates that the chemical structure of salt anion significantly affects the solvation behavior of Li^+^ and Na^+^ cations.

One may note that sodium‐based electrolytes show slightly lower viscosity for a given salt anion than the corresponding lithium samples (e.g., 1.72 ± 0.01 cP (NaPF_6_) vs. 1.82 ± 0.03 cP (LiPF_6_), Figure 2a). In general, Na^+^ cation exhibits a larger Shannon ionic radius and lower charge density, and thus weaker ion‐pairing, leading to relatively loose and weakened solvation sheaths. Therefore, the solvation sheath of Na^+^ cation is expected to be dynamic and readily undergoes spatial changes under external force (e.g., Stokes radius in propylene carbonate: 460 pm (Na^+^) vs. 480 pm (Li^+^)).^[^ ^37^ ^]^

Figure 2b further compares the ionic conductivity (*δ*) of sodium‐ and lithium‐based electrolytes. LiPF_6_‐based electrolyte exhibit the highest ionic conductivity among the lithium samples (7.10 ± 0.22 mS cm^−1^), followed by LiTFSI (6.49 ± 0.14 mS cm^−1^) > LiClO_4_ (5.07 ± 0.01 mS cm^−1^) > LiOTf (1.54 ± 0.02 mS cm^−1^). A similar trend is observed for sodium‐based electrolytes, i.e., 6.46 ± 0.17 mS cm^−1^ (NaPF_6_) > 5.87 ± 0.26 mS cm^−1^ (NaTFSI) > 4.89 ± 0.09 mS cm^−1^ (NaClO_4_) > 2.44 ± 0.21 mS cm^−1^ (NaOTf). It is interesting to note that lithium‐based electrolytes with PF_6_^−^ and TFSI^−^ anions are more conductive than the respective sodium ones, while LiOTf‐based sample shows lower ionic conductivity than NaOTf sample.

Walden analysis has been carried out to get an in‐depth understanding of the transport patterns in the sodium‐ and lithium‐based electrolytes (Figure 2c). To evaluate the degree of ion dissociation in these ionic solutions, the concept of ionicity, suggested by Angell et al.^[^ ^38^, ^39^, ^40^, ^41^ ^]^ (as described in Equation (1)), is implemented.

(1) $$\%\;\text{Ionicity}=-10^{\Delta W}\times 100\%.$$

where Δ*W* is the vertical distance measured from the ideal line of the KCl solution in the Walden plot.^[^ ^42^ ^]^

For lithium‐based electrolytes, the ionicity decreases in the order of LiTFSI (7.85%) > LiPF_6_ (7.67%) > LiClO_4_ (5.95%) > LiOTf (2.52%), in which such a trend is also noted in other lithium‐based electrolyte systems.^[^ ^43^, ^44^, ^45^, ^46^, ^47^, ^48^, ^49^, ^50^ ^]^ This suggests that the affinity of the salt anion toward Li^+^ cation increases in the order of TFSI^−^ < PF_6_^−^ < ClO_4_^−^ < OTf^−^. Following a similar pattern, the ionicity of sodium‐based electrolytes decreases in the order of NaTFSI (6.99%) > NaPF_6_ (6.68%) > NaClO_4_ (5.55%) > NaOTf (3.32%). One may note that for TFSI^−^, PF_6_^−^, and ClO_4_^−^ systems, the ion pair association of the sodium samples is significantly lower than the corresponding lithium ones. As an exception, NaOTf‐based sample exhibits a higher ionicity compared to its LiOTf counterpart. This is because Na^+^ exhibits weaker interactions with OTf^−^ compared to Li^+^, allowing more free ions to contribute to ionic conduction.

**Figure 3:**
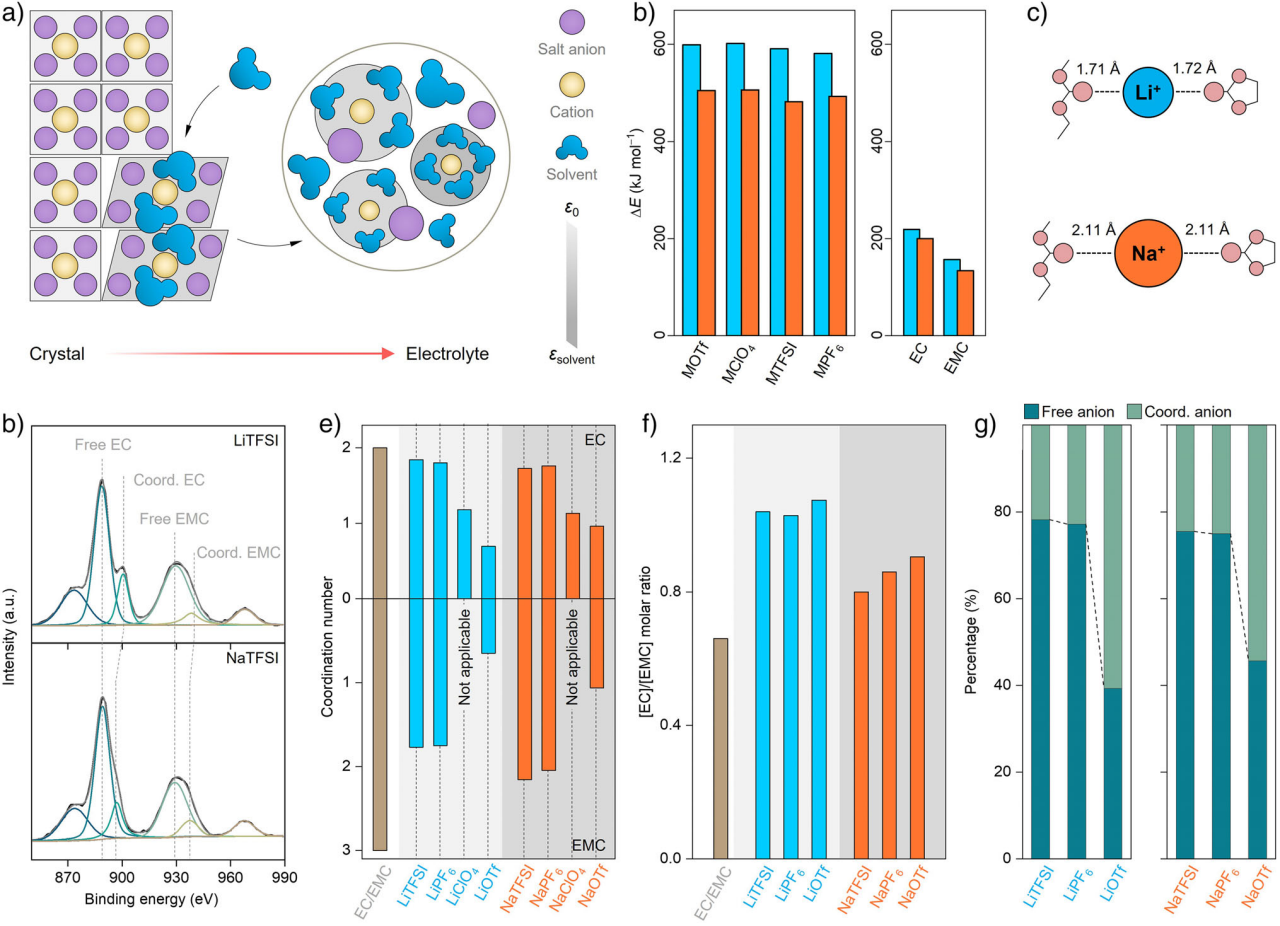
Solvation structures of sodium‐ and lithium‐based electrolytes. a) Sketch for the solvation of conducting salt in nonaqueous electrolytes. b) Energy evolution of the formation of [cation]_1_–[anion]_1_ and [cation]_1_–[solvent]_1_. c) Distance between cation and solvent in [cation]_1_–[solvent]_1_, calculated by DFT. d) Raman bands associated with C‒O vibration (EC and EMC) in LiTFSI‐ and NaTFSI‐based samples. e) Estimated coordination number of EC and EMC for lithium‐ and sodium‐based samples. f) Molar ratio of [EC]/[EMC] in the solvating sheath of metal cations. g) Proportion of free anion and coordinated anion for the lithium and sodium samples.

In general, separating an ion pair by solvent (i.e., solvation) could be regarded as a competitive coordination between salt anion and solvent for a given cation (Figure 3a). Firstly, the interaction between the cation–anion (ion–ion) pair and cation–solvent (ion‐dipole) pair is assessed by density functional theory (DFT) calculations (Tables S2 and S3). Figure 3b,c exhibits the energy required to separate two types of ion pairs (i.e., [cation]_1_–[anion]_1_ and [cation]_1_–[solvent]_1_) and [cation]–[solvent] distance, respectively. In gas phase, the dissociation energies (Δ*E*_d_) of the [Li]_1_–[anion]_1_ samples decrease in the order of [Li]_1_[ClO_4_]_1_ (602 kJ mol^−1^), [Li]_1_[OTf]_1_ (599 kJ mol^−1^) > [Li]_1_[TFSI]_1_ (591 kJ mol^−1^) > [Li]_1_[PF_6_]_1_ (581 kJ mol^−1^), in which the anions with better delocalized negative charges enable relatively weaker interactions with lithium cation (e.g., TFSI^−^ vs. OTf^−^).^[^ ^51^, ^52^ ^]^ Specifically, the Δ*E*_d_ values are systematically higher than those of the [Li^+^]_1_–[solvent]_1_ pair (Figure 3b). For sodium‐based electrolytes, the interaction strength of Na^+^ cation with either salt anion or solvent decreases as compared with the respective lithium samples (Figure 3b) due to the lower charge density of the former (i.e., 36 C mm^−3^ (Na^+^) vs. 87 C mm^−3^ (Li^+^)).^[^ ^53^ ^]^

Raman spectroscopy is employed to characterize the solvation structures of the two families of electrolytes.^[^ ^46^, ^47^, ^48^ ^]^ The Raman band associated with the stretching vibration mode of the C─O bond (850‒950 cm^−1^) is selected to determine the average coordination number (CN) of carbonate solvents toward metal cations since such region has been identified as a sensitive domain for quantitatively understanding the ion–dipole interactions in nonaqueous electrolytes (Figures S2, S3 and Table S4).^[^ ^54^ ^]^ As a general trend, the electron donicity (also known as electron donor ability) of aprotic solvents plays an essential role in the solvation processes of metal cations, and their dielectric properties are more relevant to the separation of charged ions. For both EC and EMC solvents, the carbonyl group generally exhibits moderate donicity toward metal cations,^[^ ^55^, ^56^, ^57^, ^58^, ^59^ ^]^ while the former one holds a stronger capability of separating charges due to its high dielectric constant (89.2 (EC) vs. 2.9 (EMC), Figure 3a).^[^ ^60^ ^]^

Figure 3d compares the Raman spectra of lithium‐ and sodium‐based electrolytes using TFSI^−^ anion in the selective region, and the raw data with a full scan is provided in Figure S2. Peaks in the Raman shift of ∼890 and ∼930 cm^−1^ are associated with the “free” EC and EMC molecules, respectively; and peaks at ∼900 and ∼940 cm^−1^ are associated with the “bound” EC and EMC molecules, respectively (Figure 3d).^[^ ^54^ ^]^ The calculated CNs are collectively shown in Table S5. It is noteworthy to note that the calculation of the average CNs of EMC to LiClO_4_ is not possible due to the overlap of the stretching band of ClO_4_^−^ anion and the C─O single bond in EMC (cf. Table S4).

In general, a CN value of [Li^+^]/[solvent] < 4 is noticed for the lithium samples, indicating the presence of a contact ion pair (CIP) or ionic aggregates (AGG) to a certain extent (see Figure 3e; cf. Supporting Discussion #2). The CN values decrease in the order of LiTFSI (3.61) > LiPF_6_ (3.55) > LiOTf (1.35). A similar trend is noted in sodium‐based electrolytes (e.g., NaTFSI (3.88) > NaPF_6_ (3.81) > NaOTf (2.05)), which is rationalized by the fact that the affinity of salt anions toward cations is mainly related to their inherent properties. For a fixed salt anion, the CN value of the sodium sample is higher than that of its lithium analogue. This would be mainly associated with the larger size of Na^+^ cation together with its weaker cation–solvent interactions (Figure 3b,c), which is further demonstrated by DFT calculations (see Figure S4; cf. Supporting Discussion #2).

For lithium‐based samples, the molar ratio of [EC] to [EMC] in the solvation sheath of Li^+^ cation is approximately 1.05, irrespective of the identity of the salt anion, which is significantly higher than that of the EC‐EMC (3:7, v:v) solvating media (i.e., [EC]/[EMC] = 0.66, Figure 3f and Table S5). A similar trend is also observed for the sodium system (Figure 3f). This suggests that EC is more predominant over EMC in solvating both Li^+^ and Na^+^ cations, which is related to its more planar spatial geometry and higher polarity compared to the linear EMC molecules.^[^ ^60^ ^]^ This is further manifested by DFT calculations, in which the stabilization energy of the four‐fold cation–solvent coordinating systems increases monotonically by replacing EMC with EC, e.g., −363 kJ mol^−1^ ([Na]_1_[EMC]_4_) vs. −403 kJ mol^−1^ ([Na]_1_[EMC]_2_[EC]_2_) vs. −427 kJ mol^−1^ ([Na]_1_[EC]_4_), as shown in Table S3. It is interesting to note that the [EC]/[EMC] ratios for sodium‐based samples are systematically lower as compared to those for the corresponding lithium analogue (Figure 3f). The higher proportion of EMC in the first solvation sheath of Na^+^ cations indicates that, as compared with the lithium ones, the impact of the spatial geometry of solvents on competitive coordination (i.e., solvation) is reduced in sodium electrolytes, attributing to the larger size of Na^+^ cation and its weaker cation–solvent interactions. The enrichment of EMC in the first solvation sheath impairs the power of solvent media in separating the cation–anion pair as compared to the EC‐rich ones, due to the lower dielectric constant of the linear carbonate (2.9 (EMC) vs. 89.2 (EC) at 25 °C, Figure 3a). As a consequence, the degree of ion associations tends to be more pronounced for TFSI^−^, PF_6_^−^, and ClO_4_‐based samples when paired with Na^+^ cations versus Li^+^ cations (cf. Figure 2d), in which large differences of EC‐EMC ratios are noticed among the corresponding sodium and lithium samples. The increased ion association is responsible for the slightly lower ionic conductivities observed for sodium electrolytes using these three kinds of salt anions (cf. Figure 2b).

Notably, the [EC]/[EMC] ratio is much comparable for OTf‐based samples (e.g., NaOTf (0.91) vs. LiOTf (1.07), Table S5). In this case, Li^+^ cations, which are featured with higher charge density, tend to strongly interact with OTf^−^ anion to form ion pairs, thus leading to lower ionic conductivities (cf. Figure 2b).

Characteristic signals in the Raman shift of 700–750 cm^−1^ allow the differentiation of “bound” and “free” salt anions, and thus to study their coordination situation (Figure S5 and Table S5). ClO_4_‐based samples are excluded because their most active Raman band overlaps with EMC, as mentioned above. For lithium‐based electrolytes, the proportion of “free” salt anion increases in the order of LiOTf (39.3%) < LiPF_6_ (77.2%) < LiTFSI (78.2%) A similar phenomenon is also observed for sodium‐based samples (Figure 3g), i.e., NaOTf (45.7%) < NaPF_6_ (75.0%) < NaTFSI (75.5%). Interestingly, the ion pairs with fixed salt anions show obvious differences in the dissociation ability between the two cation chemistries. For example, the exclusion of TFSI^−^ anions from the solvating environment of metal cations is somewhat energetically favorable for lithium‐based systems than that of sodium analogs (i.e., the proportion of “free” salt anion: 78.2% (LiTFSI) vs. 75.5% (NaTFSI), Figure 3g). In stark contrast, an opposite tendency is noticed when the affinity of salt anion toward metal cation is increased, particularly for the OTf‐based ones, e.g., 45.7% (NaOTf) versus 39.3% (LiOTf) for the proportion of “free” salt anion (Figure 3g). As discussed above, this could be mainly related to the increased proportion of EC in the solvation environment.

In brief, a larger volume and lower charge density of sodium cation contribute to a lower interaction strength with the salt anion/solvent and larger solvation sheaths, which enable suppressed spatial selectivity of solvent molecules, i.e., less sensitivity toward the solvent change from cyclic carbonate EC to linear EMC molecules (cf. Figure 3f). These characteristics of Na^+^ cation allow more active participation of EMC molecules in the solvation process, which reduces the ability of the solvation environment to separate ion pairs (cf. Figure 2d). The solvation sheaths of sodium cations tend to be looser than those of lithium cations, facilitating their deformation (i.e., lower viscosities of the sodium‐based electrolytes, cf. Figure 2b) and inhibiting the close packing of sodium solvates (i.e., sluggish crystallization kinetics, cf. Supporting Discussion #1).^[^ ^61^, ^62^ ^]^

### Temperature‐Induced Chemical Stability

The chemical stability of nonaqueous electrolytes is of vital importance for the long‐term cycling and safety of both sodium‐ and lithium‐based rechargeable batteries.^[^ ^63^ ^]^ To quickly assess their temperature‐induced chemical stability, the as‐prepared sodium‐ and lithium‐based electrolytes were subjected to thermal storage at 80 °C for 14 days, a test procedure that is widely used in the LIB electrolyte domain (cf. Supporting Discussion #3).^[^ ^64^, ^65^, ^66^, ^67^, ^68^, ^69^, ^70^ ^]^

**Figure 4:**
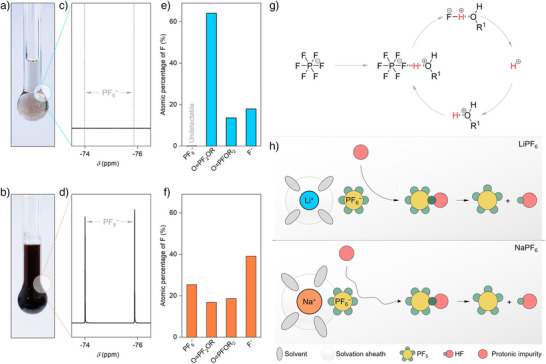
Temperature‐induced chemical stability of the sodium and lithium electrolytes. a)–b) Optical photo of the LiPF_6_ (a) and NaPF_6_ (b) electrolyte after thermal storage. c)–d) ^19^F NMR of the LiPF_6_ (c) and NaPF_6_ (d) electrolyte after thermal storage. e)–f) Atomic percentage of fluorine‐containing species in the LiPF_6_ (e) and NaPF_6_ (f) electrolyte after thermal storage. g) Schematic diagram of the chemical decomposition mechanism of the PF_6_^−^ anion proposed in Refs. [71, 72]. h) Schematic diagram of the chemical decompositions of LiPF_6_ and NaPF_6_ electrolytes.

Figure 4a,b show the digital images of the appearance of LiPF_6_‐ and NaPF_6_‐based electrolytes, respectively, and those for the electrolytes with other salts are presented in the Supporting Information. Transparent and colorless liquids are observed for both kinds of electrolytes before thermal storage (Figure S6). After being kept at 80 °C for 14 days, the LiPF_6_‐based electrolyte turns pale yellow (Figure 4a), while the NaPF_6_‐based one exhibits a darker yellow‐brown color (Figure 4b). Regardless of the intensity of the change, these observations suggest that both samples undergo chemical degradations during thermal storage. Figure 4c,d present the ^19^F NMR spectra of NaPF_6_ ‐ and LiPF_6_‐based electrolytes, respectively, after thermal storage (cf. Figures S7 and S8 for full spectra).

For lithium‐based electrolyte, the peaks assigned to P *F*_6_ anions (−75.89 ppm) are completely indiscernible after thermal storage (Figure 4c,e), accompanied by the appearance of a series of degradation products (Figures S7 and S9). Similarly, various kinds of degradation species are observed for NaPF_6_‐based electrolytes after thermal storage (Figures S8 and S10). However, in a glaring contrast to the LiPF_6_‐based electrolyte, ∼25 mol% of the PF_6_^−^ anions remain intact in NaPF_6_‐based electrolyte after thermal storage (Figure 4f), suggesting that the degradation of PF_6_^−^ anions is kinetically slow in the latter.

As demonstrated in our previous work,^[^ ^71^ ^,72]^ the chemical decompositions of LiPF_6_‐based electrolytes are induced/catalyzed by traces of protonic impurities (e.g., ROH, HF, H_2_O), resulting in highly reactive notorious intermediates such as PF_5_, POF_3_, and HF, which could further induce decompositions of the carbonate solvents, giving protonic (e.g., ROH and HF) and fluorinated alkyl phosphate species (O = PF_2_(OH), O = PF_2_(OCH_2_CH_3_), etc.; Figure 4g). The tendency of protonic impurities toward PF_6_^−^ anions and the electrostatic force between the cations and anions play key roles in determining the kinetics of the aforementioned processes. In general, a “naked” PF_6_^−^ anion is more susceptible to attack by protonic impurities. As demonstrated in Section 2.1, Na^+^⋯PF_6_^−^ pairs are less separated in EC‐EMC medium as compared with Li^+^⋯PF_6_^−^ pairs. Therefore, reduced kinetics and degree of reaction between the protic impurities and PF_6_^−^ anion are expected for the sodium‐based sample, thus hampering the temperature‐induced chemical decomposition (Figure 4h), which could bring a positive impact from the safety perspective (cf. Supporting Discussion #4).

For electrolytes with other salt anions (e.g., TFSI^−^, ClO_4_^−^, and OTf^−^), negligible change in appearance is observed (Figure S11) after thermal storage. For example, only characteristic peaks assigned to the ─CF_3_ moiety of the TFSI^−^ anion are observed for NaTFSI‐ (−81.07 ppm) and LiTFSI (−81.96 ppm)‐based samples after thermal storage (Figures S12 and S13), without the detection of decomposition products, in which a similar trend is observed for OTf‐based samples (Figures S14 and S15). This suggests that the inherent property of the salt anion dominates the chemical stability of these electrolytes. Therefore, a delicate selection of salt anions is of vital importance to improve the chemical stability of the electrochemical system and thus boost the long‐lifespan cycling performance and safety of the batteries.^[^ ^73^, ^74^ ^]^

### Impact on Corrosion Behavior of Aluminum Foil

For both lithium‐ and sodium‐based battery chemistries, the reversible conversion of chemical and electric energy relies solely on the redox reactions of the electroactive materials. Thus, other cell components must maintain sufficient chemical and electrochemical stabilities when exposed to electrolyte solutions.^[^ ^1^ ^]^ Aluminum foil (Al) is the most widely used current collector for both lithium‐ and sodium‐based batteries, and its electrochemical oxidation (i.e., Al^0^ → Al^3+^ + 3e^−^) readily occurs at relatively low potentials while exposing fresh Al^0^ surface to the electrolyte solution.^[^ ^75^ ^]^ It has been generally accepted that adequate passivation of aluminum at high potentials (e.g., > 4.0 V vs. Li/Li^+^) is an important step toward developing high‐energy‐density rechargeable batteries.^[^ ^75^ ^]^ This phenomenon has been extensively studied in LIBs, and results are usually directly transferred into RSB systems without specific cautions. To gain a comprehensive understanding of aluminum corrosion, aluminum foil's electrochemical behavior and stability were systematically investigated in sodium‐ and lithium‐based electrolytes using constant‐potential DC polarization, scanning electron microscopy (SEM), and X‐ray photoelectron spectroscopy (XPS).

**Figure 5:**
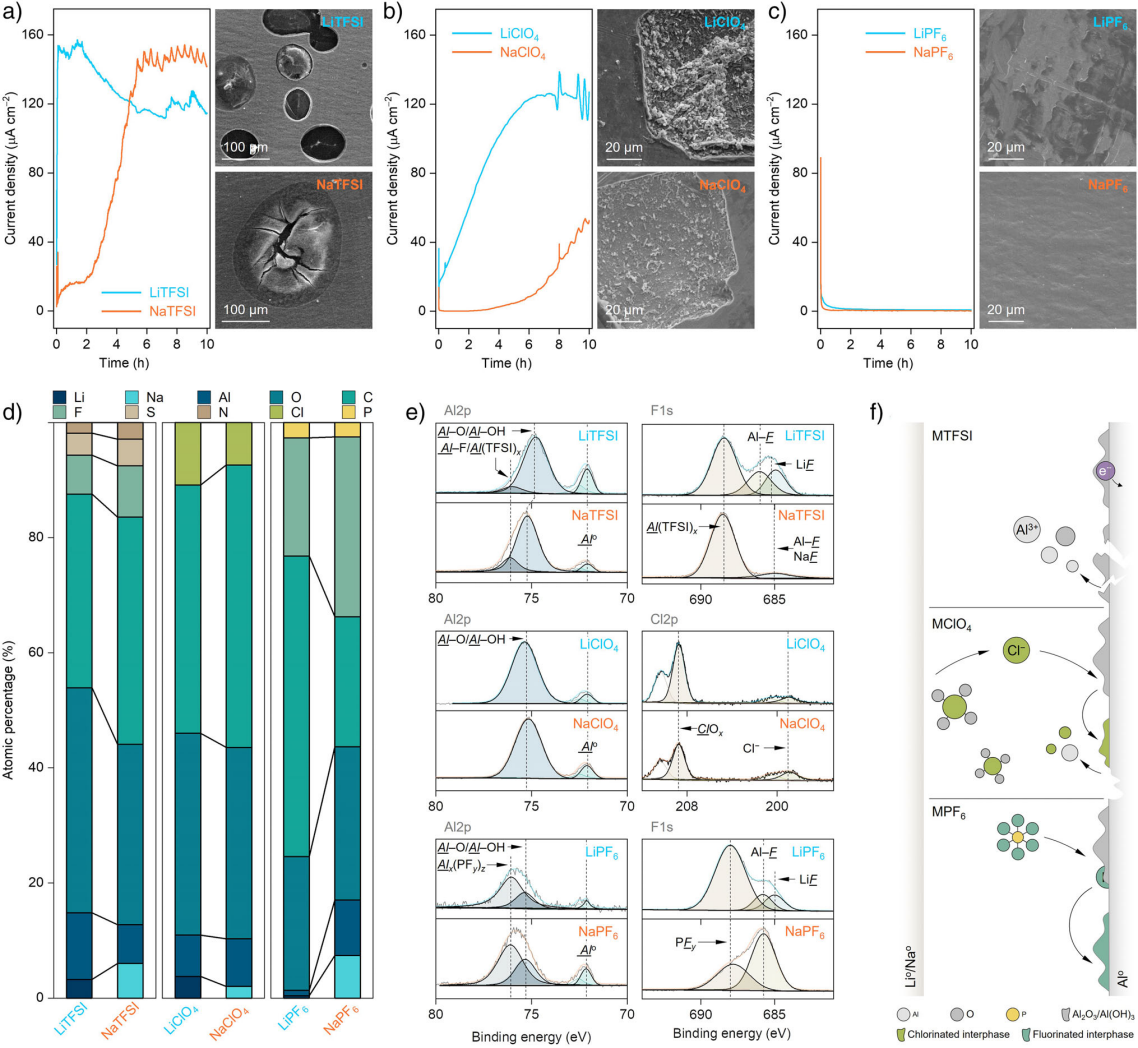
Interfacial compatibility toward aluminum foil for the sodium and lithium electrolytes. a)–c) Chronoamperometry curve and SEM images of the aluminum foils recovered from the TFSI‐ (a), ClO_4_‐ (b), and PF_6_‐ (c) based samples after DC polarization measurements. d) Normalized atomic percentage of the elements detected by XPS for the recovered aluminum foils. e) Representative XPS spectra of the recovered aluminum foils. f) Schematic illustration for the interfacial behavior of aluminum foils in the sodium and lithium electrolytes.

From the DC polarization measurements performed at a potential of 4.2 V (vs. Li/Li^+^), three types of anodic processes are generally observed for lithium‐based samples (Figure 5), namely 1) a sharp increase in current density under external polarization potential, which is a clear sign for the occurrence of the aluminum dissolution process, as also evidenced by large corrosion pits in the SEM images (i.e., LiOTf and LiTFSI, Figures 5a and S16); 2) a gradual increase in current density after the initial convergence during continuous polarization, in which surface damage is also observed in the SEM images (i.e., LiClO_4_, Figure 5b); and 3) a quick drop in current density by applying polarization potential, accompanying with long‐term stabilization at extremely low currents (<10 µA cm^−2^), indicating that aluminum maintains electrochemical stability without surface damage under the experimental condition (i.e., LiPF_6_, Figure 5c). For sodium‐based samples, the DC polarization tests were carried out at the potential of 4.0 V (vs. Na/Na^+^) to match the potential value used for the lithium‐based samples.^[^ ^18^ ^]^ A similar trend in the anodic stability of aluminum foil is noticed for the sodium samples, i.e., NaOTf < NaTFSI << NaClO_4_ << NaPF_6_. These results suggest that the identity of salt anion plays a dominating role in determining the electrochemical stability of aluminum current collector.

In general, the electrochemical stability of aluminum in nonaqueous electrolytes is linked to the solubility of complexes/salts formed between aluminum and salt anions as well as governing interfacial reactions. For anions investigated in this work, their aluminum complexes/salts are found to be mostly soluble in nonaqueous media,^[^ ^76^, ^77^, ^78^, ^79^, ^80^, ^81^, ^82^ ^]^ though clear distinctions in surface passivation of aluminum foil are noticed (Figure 5a‒c). However, several peculiar behaviors are observed for the sodium‐based samples as compared to lithium‐based ones for a fixed anion. For example, the onset of aluminum dissolution requires a longer duration in DC polarization for the sodium‐based electrolytes (e.g., 8.0 h (NaClO_4_) vs. 0.7 h (LiClO_4_) at a current density of 30 µA cm^−2^, Figure 5b). In addition, the surface of aluminum foil recovered from the NaPF_6_‐based electrolyte tends to be cleaner and smoother, in contrast to the corresponding lithium‐based one (Figure 5a). These observations indicate that the identity of the cation also notably impacts the electrochemical behavior and stability of the aluminum foil.

XPS analyses were conducted to gain deeper insight into the electrochemical stability of aluminum in the as‐prepared electrolytes. Figure 5d comparatively displays the atomic percentage of elements detected on the surface of recovered aluminum foil. For lithium‐based samples, the atomic percentage of aluminum and oxygen decreases with suppressed corrosion currents (e.g., Al: 23.4% (LiOTf) > 20.1% (LiTFSI) > 19.9% (LiClO_4_) > 1.5% (LiPF_6_)), while the species derived from salt anions become enriched (e.g., F‐contained species for LiOTf, LiTFSI, and/or LiPF_6_ samples; Cl‐contained species for the LiClO_4_ sample, Figure 5d). Similar trends are noticed for the sodium‐based electrolytes. These observations suggest that the interfacial behavior of the salt anion is of vital importance in governing the electrochemical stability of aluminum at high potentials.

In the high‐resolution XPS spectra (Figures 5e and S17), characteristic peaks associated with the salt anion are detected for all the samples, i.e., C *F*_3_‒ at 688.5 eV for TFSI‐ and OTf‐based samples, *P* F_6_ at 688.5 eV for PF_6_‐based samples, and *Cl* O_4_ at 209.5 eV. These adsorbed salt anions may interact with the active Al^3+^ cations which are possibly exposed from the electrochemical breakdown of native surface components (e.g., Al_2_O_3_ and/or Al(OH)_3_). For PF_6_‐based samples, ion pairs formed between PF_6_^−^ anions and Al^3+^ cations are relatively unstable and may undergo chemical decompositions to generate AlF_3_ and other related insoluble species on the surface of aluminum metal (e.g., Al *F*_3_ at 686.0 eV, cf. *Al* F_3_ at 76 eV, Figure 5e).^[^ ^83^ ^]^ These fluorinated surface species are electrochemically resistive toward oxidation, thus effectively protecting the fresh aluminum metal from anodic dissolution, as schematically shown in Figure 5f. The passivation ability of PF_6_‐based samples remains effective in both sodium‐ and lithium‐based electrolytes. In a stark contrast, ion pairs comprising TFSI^−^ and OTf^−^ anions and Al^3+^ cations are chemically stable (i.e., devoid of AlF_3_) and also readily soluble in carbonate solvents,^[^ ^84^, ^85^ ^]^ which leads to continuous exposure of fresh aluminum to electrolyte components and consequently severe corrosion behavior is observed (Figure 5f).

The unique feature of ClO_4_‐based electrolytes calls particular attention to the possible cross‐talk between metal anodes and aluminum current collectors. Generally, ClO_4_‐based samples are less corrosive toward aluminum current collector as compared to the TFSI‐ and OTf‐based samples (i.e., mossy structure vs. corrosive pits; Figure 5b), which is ascribed to the lower solubility and chemical stability of the complexes formed by Al^3+^ cations and ClO_4_^−^ anions, coupled with lesser selecitivity of ClO_4_^−^ versus TFSI^−^ anions toward metal surface adsorption.^[^ ^86^ ^]^ Yet, ClO_4_^−^ anion in the highest valence state of Cl (VII) may get reduced at the anode side, forming metal chloride salts (e.g., LiCl and NaCl) which are sparingly soluble in carbonate solvents. As demonstrated by the solubility tests, the maximum solubility of sodium chloride is significantly lower than that of its lithium analogue, i.e., 30.72 (LiCl) versus 0.40 (NaCl) mg kg^−1^_EC‐EMC_. In this sense, chloride (Cl^−^) ions generated in lithium‐based electrolytes may rapidly diffuse in large amounts to the surface of aluminum metal, thus triggering and accelerating its anodic dissolution process (Figure 5f). This is well supported by the enhanced resistance of aluminum foil toward anodic dissolution in sodium‐based electrolytes utilizing ClO_4_^−^ anions.

### Inspirations for Future Sodium‐Based Electrolytes and Beyond

**Figure 6:**
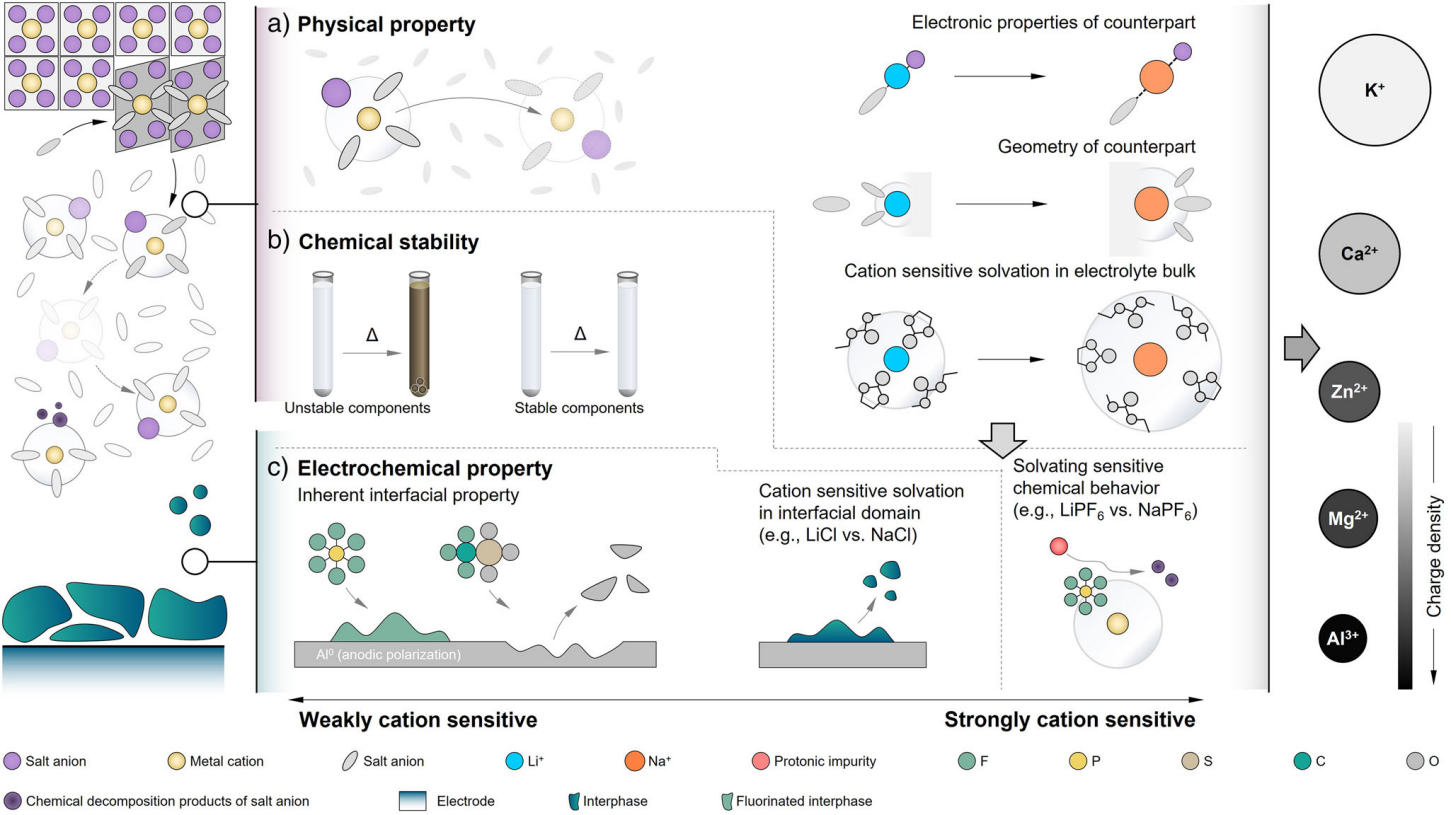
Design of sodium‐based and other related electrolytes deviating from classic lithium chemistry. a) Transport and other physical properties. b) Chemical properties of electrolyte solutions. c) Interfacial behavior and related electrochemical properties. The inherent features of sodium‐based electrolytes are well linked with their lithium analogs, while the sensitivity of cation toward the solvation process brings clear distinctions in several aspects (e.g., transport, chemical and electrochemical characteristics, etc.). This becomes more evident for other kinds of nonaqueous electrolytes containing mono‐valent (e.g., K^+^) and multivalent (Zn^2+^, Ca^2+^, Mg^2+^, and Al^3+^) cations.

Built on the copious research activities dedicated to lithium‐based electrolytes, the development of sodium‐based systems has greatly accelerated in the past years.^[^ ^11^, ^12^ ^]^ From the above systematic investigations involving some representative sodium‐ and lithium‐based electrolytes, certain conscious inspirations could be adopted to screen future electrolyte candidates for sodium batteries (Figure 6).

For ion transport and related physical properties, the monovalent charge carried by either Na^+^ or Li^+^ cation plays a minor impact on the determination of microscopic bulk transport behavior, and most of the transport patterns observed in lithium‐based electrolytes could be transferred into sodium‐based systems, e.g., motion of solvated species in electrolyte with moderate concentrations (Figure 6a).^[^ ^7^, ^8^, ^9^ ^]^ However, the better dispersion of positive charge in Na^+^ cations (i.e., lower charge density) allows the employment of solvents with larger size and salt anions with lesser degree of negative charge delocalization for reaching decent ionic conductivities, as noted by the enhanced transport for OTf‐based electrolyte in sodium systems.

For chemical properties, the solvation structures of sodium cations turn out to be critical for certain scenarios (Figure 6b). In the binary mixture of EC and EMC, the higher proportion of EMC in the solvating environment of Na^+^ cations enables the inclusion of anions in the first solvation sheaths. This may protect the anion from direct attack by protonic impurities, thus leading to better chemical stability for the sodium‐based electrolytes under long‐term storage (Figure 6b). Moreover, weaker ion‐pairing and lower Lewis acidity of Na^+^ lead to lesser destabilization power on the PF₆⁻ anion and thus limiting the thermal decomposition of NaPF₆ into NaF and PF₅ at elevated temperatures. This further brings a positive impact from the safety perspective, including delaying the onset of the SEI decomposition and thus avoiding further cascading electrolyte reduction and ultimate thermal runaway. This is also beneficial from a thermal and chemical (i.e., fire‐induced toxicity) perspective during electrolyte combustion and fires under oxygen‐rich and lean environments, as such safety appraisal is critical to building durable batteries.

With regard to electrochemical properties, the distinctive solvation structures possessed by the sodium‐based electrolytes may significantly impact the solubilization and transport of intermediate species formed in electrochemical reactions (Figure 6c). As reflected by the aluminum corrosion tests, the interfacial species generated from the electrochemical reductions of ClO_4_^−^ anions on alkali metal electrodes tends to be less soluble for the sodium‐based electrolytes, which contributes to better passivation of aluminum current collector with its native film in the NaClO_4_‐based electrolyte at higher potentials (Figure 5b).

In brief, while the knowledge and experience gained from lithium‐based batteries provide a crucial foundation, the design of electrolytes for advanced sodium and other emerging battery systems must consider the unique chemistry of the cations and their interactions with the counter anions. These systems should move beyond mere mimicry of lithium‐based designs and instead embrace innovative and out‐of‐the‐box approaches.

## Conclusion

In this work, the fundamental properties of a series of sodium and lithium electrolytes are systematically studied, and the following conclusive remarks could be adopted:

1. A larger volume and lower charge density of sodium cations contribute to a lower interaction strength between metal cations and salt anions (ion–ion)/solvent (ion‐dipole). Therefore, the solvation sheaths of sodium cations tend to be larger and looser than those of lithium cations, facilitating their deformation (i.e., lower viscosities) and inhibiting the close packing of sodium solvates (i.e., sluggish crystallization kinetics).
2. The solvation environments of alkali metal cations formed in mixed solvents (e.g., EC‐EMC) are cation‐sensitive, which results in uncoordinated properties between the lithium and sodium systems (e.g., ion dissociation and temperature‐induced chemical stability).
3. The noncorrosive features of PF_6_^−^ anion are inherited from lithium to sodium‐based electrolytes; while the ClO_4_‐based samples show strong sensitivity toward the identity of metal cation, in which the sodium cation presented a decreased anodic dissolution of aluminum current collector at high potentials, due to the reduced solubility of chloride‐containing sodium salts (e.g., NaCl) generated from the reductive decomposition of ClO_4_^−^ anions at the anode side, compared to its LiCl counterpart.

Overall, this work provides not only an invaluable reference for the evaluation of state‐of‐the‐art sodium‐based electrolytes but also serves as a guiding principle toward the screening, design, and development of function‐oriented electrolytes for advanced sodium, and other emerging mono‐ (e.g., potassium) and multivalent (e.g., calcium, magnesium, aluminum, etc.) cation‐based electrolytes.

## Conflict of Interests

The authors declare no conflict of interest.

## Supporting information



Supporting Information

## Data Availability

The data that support the findings of this study are available from the corresponding author upon reasonable request.
