# Supplementary material for: Electrolyte Chemistry Development for Sodium‐Based Batteries: A Blueprint from Lithium or a Step Toward Originality?
Source: Angew Chem Int Ed Engl. 2025 Apr 14;64(23):e202424543. doi: 10.1002/anie.202424543 (PMC12124431; doi:10.1002/anie.202424543)
Supplement: Supplementary file 1 — Supporting Information [file ANIE-64-e202424543-s001.pdf]

Supporting Information

Electrolyte Chemistry Development for Sodium-Based Batteries: A Blueprint from Lithium or a Step Toward Originality?

Ziyu Song,[a]+ Zhirong Xing,[a]+ Jiaxun Yang,[a] Jiayi Chen,[a] Weican Hu,[a] Pu Li,[a] Wenfang Feng,[a] Gebrekidan Gebresilassie Eshetu,*[b,c] Egbert Figgemeier,[b,d] Stefano Passerini,[e] Michel Armand,[f] Zhibin Zhou,[a] and Heng Zhang*[a]

+ Both authors contributed equally to this work. This work is dedicated to Professor Bruno Scrosati, a pioneer in alkali-ion batteries and a great mentor, colleague, and friend.

[a] Z. Song+, Z. Xing+, J. Yang, J. Chen, W. Hu, P, Li, Prof. Dr. W. Feng, Prof. Dr. Z. Zhou, Prof. Dr. H. Zhang
Key Laboratory of Material Chemistry for Energy Conversion and Storage (Ministry of Education), School of Chemistry and Chemical Engineering
Huazhong University of Science and Technology
Wuhan 430074, China
E-mail: hengzhang2020@hust.edu.cn

[b] Dr. G. G. Eshetu, Prof. Dr. E. Figgemeier
Institute of Power Electronics and Electric Drives (ISEA)
Center for Ageing Reliability and Lifetime Prediction of Electrochemical and Power Electronic Systems (CARL)
RWTH Aachen University
Campus Boulevard 89, 52074 Aachen, Germany
E-mail: Gebrekidan.Eshetu@isea.rwth-aachen.de

[c] Dr. G. G. Eshetu
Department of Material Science and Engineering, Mekelle Institute of Technology
Mekelle University
P.O. Box 1632, Mekelle, Ethiopia

[d] Prof. Dr. E. Figgemeier
Helmholtz-Institute Münster (HI MS) IMD-4
Forschungszentrum Jülich
46, 48149 Münster, Germany

[e] Prof. Dr. S. Passerini
Austrian Institute of Technology (AIT), Center for Transport Technologies
Giefinggasse 4, 1020, Wien, Austria

[f] Prof. Dr. M. Armand
Centre for Cooperative Research on Alternative Energies (CIC energiGUNE)
Basque Research and Technology Alliance (BRTA)
Vitoria-Gasteiz 01510, Spain

1. Supplementary Methods

1.1. Theoretical Approaches. Density functional theory (DFT) calculations were performed using the software developed by Fritz Haber Institute, namely Fritz Haber Institute ab initio molecular simulations (FHI-aims)[1]. To generate initial input files of the geometries, the Avogadro software was used[2]. More computational details could be found in our previous work[3].

1.2. Materials. Anhydrous and high-purity (> 99.9%) lithium (Li) and sodium (Na) conducting salts were obtained from commercial sources: LiTFSI (Solvey, Belgium), NaTFSI (Solvey, Belgium), $LiPF_6$ (Morita Chemical, Japan), $NaPF_6$ (Sigma-Aldrich, USA), $LiClO_4$ (Aladdin, China), $NaClO_4$ (Aladdin, China), LiOTf (Aladdin, China), NaOTf (Aladdin, China). The carbonate solvents [EC (>99%, TCI, Japan) and EMC (>99%, Sigma-Aldrich, USA)] were purified using standard procedures[4]. The lithium and sodium electrolytes were prepared by dissolving conducting salt in EC-EMC (3:7, v/v) in a volumetric flask. The water contents were found to be lower than 30 ppm (Karl-Fischer titration, Metrohm KF831).

1.3. Physical Characterizations. The phase behaviors of the lithium and sodium electrolytes were characterized by differential scanning calorimetry (DSC, Netzsch 200 F3) at a heating rate: 10 K min^{-1}. Ionic conductivities of the lithium and sodium electrolytes were measured by electrochemical impedance analysis of Pt||Pt conductance cells (cell constant: ca. 10 cm^{-1}). The electrochemical impedance spectra were recorded from 10^6 to 1 Hz on an electrochemical workstation (Multi Autolab/M204, Metrohm). The viscosities of the lithium and sodium electrolytes were measured on a programmable viscometer (Brookfield, DVIII+) at 25 °C, and the temperature was controlled with a thermostatic oil-circulator bath (± 0.1 °C, Brookfield TC-502). Raman spectra of the neat solvent and lithium- and sodium-based electrolytes were recorded with a laser confocal Raman spectrometer (LabRAM HR800, Horiba-JobinYvon). The samples were sealed in glass tubes and the incident laser with a wavelength of 532 nm was selected.

1.4. Chemical stability. The temperature-induced chemical stabilities of the lithium and sodium electrolytes were studied following the procedures described in our previous works[5]. Specifically, the electrolyte samples were flame-sealed in pressure-resistance glass tubes and were stored at 80 °C for 14 days. The compositions of the aged lithium and sodium electrolytes were analyzed by nuclear magnetic resonance (NMR) spectroscopy using a spectrometer (Bruker AV400). Coaxial NMR tubes were employed to prevent the samples from directly contacting the deuterium reagent (i.e., acetone-d_6).

1.5. Anodic stabilities of aluminum foil. The chronoamperometry tests were carried out on an electrochemical workstation (Multi Autolab/M204, Metrohm) using a homemade three-electrode cell. After the test, the aluminum foil was recovered, washed by EMC, and dried under vacuum. The surface morphologies of the recovered aluminum foil were imaged by scanning

electron microscopy (SEM; SU8010, Hitachi), and the surface components were detected by X-ray photoelectron spectroscopy (XPS; AXIS SUPRA+, Shimadzu). The solubilities of LiCl and NaCl in EC-EMC (3:7, v/v) were determined by ion chromatography (881 Compact IC pro, Metrohm). Specifically, the metal chloride salts (1 g) were mixed with the solvent (10 g) at 25 °C for 24 h. The supernatant of these supersaturated solutions was filtered, and the solvent was removed under vacuum. The as-obtained solid was then washed with deionized water (100 g) to perform ion chromatography (IC, 883 Metrohm) tests.

2. Supplementary Discussions

2.1. Supplementary Discussion #1. For lithium-based electrolytes, three kinds of phase transition behaviors enlisting glass transition (T_g), cold crystallization (T_c), and melting (T_m), are observed during the heating scan (Figure S1 and Table S1). Taking LiTFSI/EC-EMC as an example, the glass transition is located far below −100 °C, and the supercooled amorphous phases undergo cold crystallization (i.e., T_{c1} and T_{c2}) at < −50 °C, Figure S1a). The crystalized solvents (i.e., EC and EMC) and their eutectic mixtures, together with the as-formed salt-solvent complexes, become fully melted before reaching the ambient temperature region, in which the endmost melting transition (i.e., T_{m3} in Figure S1a), adjacent to obtaining a complete liquidus phase, is generally associated with the melting processes of salt-solvent complexes.[6] For a fixed cation, identity of the salt anion is found to influence the T_{m3} values of the electrolyte systems [e.g., 16.2 °C ($LiPF_6$) vs. 15.0 °C ($LiClO_4$) vs. 10.6 °C (LiTFSI), Figure S1a and Table S1].

In general, an anion with a regular shape and higher radius ratio with Li^+ cation (r_{cation}/r_{anion}) is more likely to form solvated complexes with higher melting points, especially those with octahedral symmetry (e.g., PF_6^-, AsF_6^-, SbF_6^-, etc.). For example, the 2:1 monoglyme (G1)-lithium complex with tetrahedral ClO_4^- (radius ratio: 0.282) is reported to crystallize into a monoclinic *C*2/*c* space group, indicating a distinct structural arrangement that contributes to thermal properties.[7] The melting temperature of $(G1)_2LiClO_4$ is detected at ~70 °C, which is significantly higher than that of the $(G1)_2LiX$ complex (where X is an anion) with a larger $TFSI^-$ anion (radius ratio: 0.216, T_m: ~20 °C) that exhibits a monoclinic symmetry (*Cc* space group).[8] For the LiOTf-based sample, stronger ion association (*cf.* Figure 2c) results in a low proportion of solvent in the crystalline complex, thus a higher value of T_{m3} is observed (Figure S1 and Table S1)[9].

Of prime importance, the identity of the cation (Li^+ vs. Na^+) greatly influences the phase transition behavior of the resulting electrolytes. For systems with smaller salt anions (i.e., PF_6^- and ClO_4^-), sodium-based electrolytes show more sluggish crystallization and melting kinetics as indicated by the broadening of the peaks and more significant thermal hysteresis [e.g., T_c^1: −85.7 °C ($LiClO_4$) vs. −65.2 °C ($NaClO_4$); Figure S1]. With an increase in anion volume (e.g., $TFSI^-$ anion), quite comparable phase transition behaviors are noted for the corresponding lithium and sodium electrolytes (Figure S1). It has to be highlighted that, for the OTf^- system, a remarkable decrease in T_{m3} value is noticed for the sodium system [i.e., −0.7 °C (NaOTf) vs. 21.2 °C (LiOTf), Table S1], which could be related to its improved ion dissociation as discussed in Figure 2c.

2.2. Supplementary Discussion #2. A four to six-fold coordination of a single Li^+ cation toward solvent molecules is usually characterized as a threshold for complete solvation, in which the corresponding electrolyte is mostly composed of solvent-separated ion pair (SSIP, Table S6). A coordination number below this value (i.e., $[Li^+]/[solvent] < 4$) generally indicates the presence of a contact ion pair (CIP) or ionic aggregates (AGG) to a certain extent (Table S6). The dependence of CN values on the type of electrolyte salt for both lithium and sodium-

based electrolytes is depicted in Figure 3e. For TFSI-based electrolyte, an average molar ratio of $[Li^+]/[solvent]$ of 3.61 (i.e., $[Li^+]/[EC] = 1.84$ and $[Li^+]/[EMC] = 1.77$, Table S5) is obtained, implying that $[Li]_1[TFSI]_1$ ion pair might not be completely solvent-separated in EC/EMC.[10]

In comparison, sodium-based electrolyte shows a slightly higher CN value vs. the lithium analog for the TFSI-based family [e.g., 3.88 (NaTFSI) vs. 3.61 (LiTFSI)], which offers a signal for a more active contribution of solvent molecules in the solvation processes for the sodium electrolytes. DFT calculations further demonstrate that a sodium cation is able to capture a higher number of solvents to build the first solvated sheath (e.g., $[Na]_1[EC]_5$ vs. $[Li]_1[EC]_4$ and $[Na]_1[EMC]_5$ vs. $[Li]_1[EMC]_4$, Figure S4). Besides, an average Na···O distance of 2.11 Å is noticed for the four-fold Na^+-EC coordinating (*cf.* Figure 3c), which is significantly larger than that of the lithium system (ca. 1.72 Å, *cf.* Figure 3c). These results could be associated with the larger size of Na^+ cation, together with its weaker cation-solvent ion-pair interactions.

As a general trend, for lithium-based electrolytes, the molar ratio of $[Li^+]/[solvent]$ increases in the order of LiOTf (1.35) < $LiPF_6$ (3.55) < LiTFSI (3.61). The increased CN values indicate that the dissociation of the conducting salt is enhanced by replacing OTf^- anion with either PF_6^- or $TFSI^-$ anions, which is consistent with the aforesaid observation of the transport properties (*cf.* Figure 2a), i.e., the ionicity increases in the order of LiOTf (2.52%) < $LiPF_6$ (7.67%) < LiTFSI (7.85%). A similar trend is noted in sodium-based electrolytes [e.g., NaOTf (2.05) < $NaPF_6$ (3.81) < NaTFSI (3.88)], which is rationalized by the fact that the affinity of salt anions toward cations is mainly related to their inherent properties.

2.3. Supplementary Discussion #3. Chemical stability is regarded as the resistance of the electrolyte constituents and polarized interphases to remain chemically unchanged under any condition, including storage at low and high temperatures, long-term cycling, battery shipping, etc. The temperature-induced chemical stability is investigated by thermal storage test, and their degradation processes were probed via nuclear magnetic resonance (NMR) measurements [11]. The fact that a relatively high temperature of 80 °C has been selected is to mimic the salt anion/solvent contributions since such value (i.e., 70–80 °C) is usually regarded as the onset temperature at which the solid electrolyte interphase (SEI) starts to decompose, and thus triggering cascaded exothermic electrolyte reduction reactions.[12] In addition, this test procedure is widely used in assessing the temperature-induced chemical stability of LIB electrolytes[13]. Herein, customized co-axial NMR tubes were employed to isolate the electrolyte sample from deuterated solvents.

2.4. Supplementary Discussion #4. As is well-known, the key ingredients of the SEI possessing high basicity (e.g., Li_2CO_3/Na_2CO_3, $ROCO_2Li/ROCO_2Na$, ROLi/RONa, etc.) are highly prone to reactions with the strong Lewis acids like PF_5 and other acidic decomposition products of $LiPF_6/NaPF_6$ (HF, POF_3, etc.) and thus drastically altering the overall composition, thickness, ion transport, electro-chemical-mechanical stability of the interphases/interfaces [Equations (S-1) and (S-2)].[14]

$$M_2CO_3\ (SEI) + PF_5 \rightarrow POF_3 + 2MF + CO_2\ (M = Li\ or\ Na) \quad (S\text{-}1)$$

$$CH_3OCO_2M\ (SEI) + PF_5 \rightarrow POF_3 + MF + CH_3F + CO_2\ (M = Li\ or\ Na) \quad (S\text{-}2)$$

Hence, the relatively high resistance of sodium-based electrolytes toward temperature-induced decomposition generates lesser amounts of acidic species that ultimately reduce the acid-base reaction of the SEI components. This could bring a positive impact from the safety perspective, including delaying the onset of the SEI decomposition and thus further cascading electrolyte reduction that leads to exothermic phenomena, and ultimately thermal runaway. Besides, the SEI layer decomposition, the impacts of the salt chemistry on the thermal and chemical (i.e., fire-induced toxicity) threats during electrolyte combustion, and fires under oxygen-rich and lean environments are of paramount importance to building durable batteries. Though a full-scale appraisal is needed, the high chemical stability and resistance to hydrolysis of $NaPF_6$ salt can lead to the release of less highly toxic and hazardous gases such as HF, POF_3, and other fluorinated phosphates compared to its $LiPF_6$ counterpart. Moreover, the relatively high preference of Na^+ vs. Li^+ cations for the linear carbonate (EMC, *cf.* Section 2.1 in the main text) implies that there will be less EMC in the bulk electrolyte to easily get volatilized and thus improving the resistance toward combustion (i.e., decreasing the ease flammability of EMC by cation trapping) as EC has lower flash point (24 °C) compared to EC (143 °C)[15]. Interestingly, the results obtained in this work complement recently reported findings on the combustion test of $LiPF_6$/$NaPF_6$ electrolyte, where the authors stated that a lower amount of toxic gases (mostly remaining in the residues after combustion) is detected in $NaPF_6$-based electrolyte compared to the $LiPF_6$ one.[16]

3. Supplementary Tables

Table S1. Phase transitions of lithium- and sodium-based electrolytes obtained by the first heating process of DSC measurement.

Sample	T_g[a] (°C)	T_{c1}[b] (°C)	T_{c2}[b] (°C)	T_{m1}[c] (°C)	T_{m2}[c] (°C)	T_{m3}[c] (°C)
LiTFSI	−124.1	−86.8	N/A	−67.8	1.2	10.2
$LiPF_6$	−123.8	−83.8	−38.8	−69.8	−0.9	16.2
$LiClO_4$	−119.1	−85.7	N/A	−66.8	−1.9	15.2
LiOTf	−125.3	N/A	N/A	−64.8	3.8	21.2
NaTFSI	−115.9	−85.6	N/A	−67.1	2.1	15.1
$NaPF_6$	−109.3	−38.2	N/A	−13.3	N/A	N/A
$NaClO_4$	−115.9	−65.2	N/A	−12.2	N/A	N/A
NaTf	−107.9	N/A	N/A	−67.7	−26.7	−0.3

[a] Glass transition temperature (T_g). [b] Crystallization temperature (T_c). [c] Melting temperature (T_m).

Table S2. Cartesian coordinates of the optimized structures using DFT calculations.

Atom	x	y	z	Atom	x	y	z
**$LiEC_1$**				**$NaEC_1$**			
Li	-4.51813	5.642193	0.937067	Na	-4.54416	5.299505	1.030873
C	-4.00878	10.35657	-1.02907	C	-4.0098	10.38577	-1.03905
C	-3.79923	10.70172	0.456275	C	-3.79921	10.7361	0.444768
O	-3.91655	9.409065	1.116749	O	-3.90751	9.450382	1.109714
H	-4.56515	11.35112	0.86708	H	-4.56698	11.38645	0.851566
H	-2.81378	11.09673	0.679315	H	-2.81591	11.14102	0.661344
O	-4.23496	8.917865	-1.00638	O	-4.23141	8.9515	-1.015
H	-3.13793	10.54236	-1.64894	H	-3.14004	10.57747	-1.65928
H	-4.88636	10.82028	-1.46684	H	-4.88765	10.85072	-1.47584
C	-4.1627	8.47444	0.22758	C	-4.15484	8.500131	0.224923
O	-4.31113	7.298356	0.522265	O	-4.29719	7.331652	0.521091
**$LiEMC_1$**				**$NaEMC_1$**			
Li	-4.56451	5.499754	0.716748	Na	-4.89956	5.534733	0.878014
C	-2.82046	4.282157	-2.51849	C	-2.7938	4.283597	-2.52505
O	-1.69071	4.658041	-1.69846	O	-1.6654	4.659135	-1.71145
H	-3.45453	5.145793	-2.70048	H	-3.43195	5.145075	-2.70586
H	-3.37878	3.481228	-2.04113	H	-3.35318	3.482757	-2.04778
H	-2.38546	3.934115	-3.44701	H	-2.36493	3.935173	-3.45662
C	-1.90016	5.127225	-0.50047	C	-1.88726	5.124327	-0.50549
O	-0.77982	5.409998	0.09083	O	-0.75383	5.407675	0.078779
C	-0.79006	5.952023	1.452709	C	-0.76749	5.945522	1.43461
C	0.645338	6.158557	1.864155	C	0.666811	6.156855	1.851371
H	-1.3008	5.233649	2.091699	H	-1.27708	5.228241	2.076494
H	-1.35002	6.885505	1.433226	H	-1.32958	6.878375	1.420199
H	1.193264	5.218916	1.85709	H	1.217026	5.2184	1.847134
H	0.669556	6.562007	2.875599	H	0.689433	6.563356	2.861678
H	1.144591	6.862893	1.202475	H	1.166654	6.859414	1.188058
O	-3.02504	5.280439	0.001709	O	-3.00345	5.269664	-0.00389
**$LiEC_3EMC_1$**				**$NaEC_3EMC_1$**			
C	-1.96587	3.349474	-3.09934	C	-1.98873	3.366312	-3.39266
C	-1.93906	4.868688	-3.29774	C	-2.50702	4.73774	-3.83489
O	-2.54657	5.364769	-2.08224	O	-3.06039	5.280621	-2.61431
H	-2.54255	5.202024	-4.1386	H	-3.30464	4.677476	-4.57168
H	-0.93944	5.28278	-3.37344	H	-1.72895	5.407345	-4.18558
O	-2.90336	3.181241	-2.01188	O	-2.67253	3.153495	-2.13696
H	-1.00856	2.940282	-2.78617	H	-0.91828	3.354261	-3.20074
H	-2.339	2.803256	-3.95884	H	-2.25303	2.559128	-4.06722
C	-3.13457	4.3575	-1.42673	C	-3.19442	4.305267	-1.70632
O	-3.79521	4.491906	-0.43231	O	-3.72043	4.448333	-0.63678
Li	-4.41977	5.487651	1.15134	Na	-4.45205	5.458176	1.282515
C	-2.28594	4.615205	4.730838	C	-2.72003	4.542328	5.399049
O	-2.81632	3.348567	4.30629	O	-2.89871	3.229752	4.841095
H	-3.08874	5.27104	5.058196	H	-3.6785	5.044896	5.501992
H	-1.73199	5.084665	3.921875	H	-2.05763	5.133604	4.771563
H	-1.62676	4.383583	5.559158	H	-2.27256	4.378938	6.372355

Table S2. Countinued.

Atom	x	y	z	Atom	x	y	z
C	-3.63587	3.355011	3.268511	C	-3.43855	3.157494	3.635197
O	-4.02468	2.122988	3.019742	O	-3.52505	1.891701	3.278434
C	-4.95142	1.90823	1.919032	C	-4.10604	1.594544	1.980414
C	-5.2078	0.423251	1.832163	C	-4.03383	0.099145	1.786558
H	-4.50312	2.305123	1.010913	H	-3.54754	2.134986	1.218635
H	-5.86209	2.466512	2.131342	H	-5.13402	1.955173	1.978603
H	-4.28584	-0.12149	1.638166	H	-3.00259	-0.24766	1.810338
H	-5.90186	0.223302	1.016755	H	-4.46056	-0.16057	0.818616
H	-5.64632	0.046156	2.753971	H	-4.5933	-0.42395	2.559488
O	-3.97243	4.353274	2.654421	O	-3.79502	4.114103	2.970511
C	-8.20733	4.879358	-0.66722	C	-8.33105	4.67166	-0.53969
O	-8.51568	5.330253	0.664011	O	-8.81089	5.281579	0.669991
H	-7.51301	4.043841	-0.63517	H	-7.7331	3.792829	-0.31109
H	-7.78098	5.691432	-1.25108	H	-7.74074	5.381027	-1.11446
H	-9.15663	4.570311	-1.08834	H	-9.22212	4.389745	-1.08819
C	-7.50342	5.718569	1.418969	C	-7.90163	5.721223	1.524707
O	-7.96	6.104781	2.591709	O	-8.51109	6.24579	2.568739
C	-6.99656	6.584993	3.569817	C	-7.68204	6.786355	3.631899
C	-7.7614	6.933009	4.823707	C	-8.60591	7.323899	4.698019
H	-6.48821	7.45082	3.148209	H	-7.04881	7.565109	3.209387
H	-6.26168	5.800045	3.739534	H	-7.04537	5.986836	4.007861
H	-8.49518	7.713485	4.631662	H	-9.24356	8.111815	4.301978
H	-7.06649	7.295822	5.580652	H	-8.01364	7.739491	5.51254
H	-8.27705	6.061526	5.222359	H	-9.23812	6.535542	5.101707
O	-6.33466	5.717007	1.071161	O	-6.6963	5.650264	1.363921
C	-4.64034	8.402931	-0.93995	C	-4.48457	8.757913	-0.96275
O	-3.66658	9.036359	-0.09184	O	-3.41709	9.294812	-0.16356
H	-5.50717	8.095042	-0.36108	H	-5.37662	8.614681	-0.35785
H	-4.2006	7.541991	-1.43683	H	-4.18398	7.815107	-1.41359
H	-4.91754	9.159281	-1.66524	H	-4.66672	9.502098	-1.72928
C	-3.20248	8.334705	0.92763	C	-3.01585	8.570688	0.869084
O	-2.31993	9.060852	1.582719	O	-2.03837	9.205592	1.484971
C	-1.68695	8.467161	2.748244	C	-1.45431	8.568702	2.652275
C	-0.74374	9.496331	3.323132	C	-0.36896	9.48148	3.169683
H	-1.1663	7.56529	2.429407	H	-1.06406	7.59747	2.351679
H	-2.467	8.185646	3.453728	H	-2.24303	8.412542	3.386591
H	0.018855	9.775489	2.598729	H	0.402406	9.637002	2.417862
H	-0.24739	9.082899	4.200673	H	0.093846	9.031787	4.04739
H	-1.2809	10.3935	3.624759	H	-0.77532	10.44977	3.455203
O	-3.5383	7.200089	1.218115	O	-3.47844	7.492344	1.194329
**$LiEC_2EMC_2$**				**$NaEC_2EMC_2$**			
C	-6.17279	0.283022	0.076134	C	-6.77994	0.69921	0.197067
C	-4.75288	0.279496	-0.50026	C	-5.59144	0.222222	-0.64351
O	-4.24	1.572461	-0.10502	O	-4.60247	1.250519	-0.41626
H	-4.73155	0.224235	-1.58623	H	-5.80683	0.18864	-1.70892

Table S2. Countinued.

Atom	x	y	z	Atom	x	y	z
H	-4.11133	-0.48627	-0.07725	H	-5.17896	-0.72721	-0.31877
O	-6.40166	1.680785	0.362372	O	-6.46501	2.08908	0.439606
H	-6.25931	-0.2715	1.007609	H	-6.85449	0.195888	1.15847
H	-6.93003	-0.0507	-0.62535	H	-7.73104	0.648505	-0.32244
C	-5.246	2.348221	0.316351	C	-5.17957	2.319355	0.140881
O	-5.12723	3.503487	0.616142	O	-4.61501	3.357703	0.347545
Li	-4.46343	5.256377	1.17121	Na	-4.14342	5.440154	1.154046
C	-4.74597	9.294632	-2.30618	C	-5.70079	9.53794	-2.4916
C	-3.82609	9.709125	-1.15339	C	-4.18128	9.705895	-2.39308
O	-3.66675	8.479123	-0.41052	O	-3.77456	8.532027	-1.65542
H	-4.27302	10.448	-0.49224	H	-3.884	10.58398	-1.82411
H	-2.84578	10.04444	-1.47475	H	-3.67665	9.698729	-3.35355
O	-5.29496	8.039973	-1.8452	O	-5.98147	8.549537	-1.47626
H	-4.21238	9.109121	-3.2356	H	-6.02679	9.141352	-3.45052
H	-5.56623	9.982645	-2.48123	H	-6.25726	10.43885	-2.25577
C	-4.58822	7.593364	-0.80437	C	-4.84435	7.959797	-1.09254
O	-4.76303	6.522897	-0.28826	O	-4.79405	7.030061	-0.33488
C	-1.19852	5.74275	3.782317	C	-0.45419	6.681596	3.4232
O	-0.58398	5.259689	2.574704	O	0.114042	5.839571	2.405586
H	-1.90561	5.01121	4.165164	H	-1.15199	6.116141	4.035884
H	-1.7064	6.685817	3.596159	H	-0.96202	7.53084	2.972762
H	-0.38101	5.882247	4.479924	H	0.387441	7.015425	4.018908
C	-1.38813	4.994949	1.55952	C	-0.72529	5.310753	1.529908
O	-0.67126	4.566349	0.540015	O	-0.04773	4.571191	0.674484
C	-1.37941	4.204674	-0.67604	C	-0.79587	3.910908	-0.3819
C	-0.34595	3.761393	-1.68383	C	0.179905	3.066141	-1.16489
H	-1.93716	5.074953	-1.0184	H	-1.25797	4.678739	-1.00124
H	-2.08679	3.412592	-0.43811	H	-1.58386	3.313062	0.072039
H	0.358522	4.561937	-1.90177	H	0.966789	3.678699	-1.6005
H	-0.84321	3.48052	-2.61162	H	-0.34843	2.560329	-1.97261
H	0.211435	2.900831	-1.31926	H	0.639587	2.311188	-0.52998
O	-2.59761	5.129521	1.575998	O	-1.93033	5.484993	1.524691
C	-7.96337	5.575716	1.469426	C	-7.86527	5.751338	1.660467
O	-7.78426	6.070897	2.810517	O	-7.73248	5.984675	3.07415
H	-7.59144	4.558244	1.389006	H	-7.51687	4.752272	1.408197
H	-7.44692	6.213276	0.756521	H	-7.30729	6.494059	1.095824
H	-9.03324	5.606055	1.299401	H	-8.92625	5.841643	1.457841
C	-6.548	6.145938	3.269114	C	-6.50601	5.972359	3.571821
O	-6.57295	6.599325	4.506962	O	-6.56547	6.193262	4.870598
C	-5.3012	6.750568	5.192048	C	-5.31013	6.221786	5.601311
C	-5.59328	7.27646	6.576719	C	-5.63643	6.480981	7.05217
H	-4.68178	7.435659	4.615024	H	-4.68535	7.005604	5.175457
H	-4.81017	5.779047	5.21736	H	-4.80925	5.265422	5.458705
H	-6.09606	8.240626	6.531076	H	-6.14718	7.434109	7.174601
H	-4.65779	7.403598	7.120431	H	-4.7134	6.510013	7.630249
H	-6.22124	6.583724	7.133682	H	-6.26863	5.69348	7.457739

Table S2. Countinued.

Atom	x	y	z	Atom	x	y	z
O	-5.54087	5.848001	2.651375	O	-5.48697	5.786682	2.931944
**$LiEC_4$**				**$NaEC_4$**			
C	-5.769	0.780928	0.220603	C	-6.07935	0.867895	-0.67436
C	-4.40082	1.041486	-0.41703	C	-4.69848	0.298114	-0.33532
O	-4.03574	2.330492	0.119787	O	-4.08178	1.368646	0.409037
H	-4.44267	1.124326	-1.50089	H	-4.08988	0.100605	-1.21517
H	-3.64081	0.322542	-0.12979	H	-4.73216	-0.58378	0.295866
O	-6.16532	2.090089	0.67901	O	-5.90813	2.282541	-0.45015
H	-5.72016	0.119353	1.082774	H	-6.86395	0.510529	-0.01088
H	-6.51635	0.421736	-0.47916	H	-6.37265	0.717215	-1.70804
C	-5.11023	2.91162	0.666936	C	-4.7812	2.498111	0.241498
O	-5.13195	4.030552	1.096721	O	-4.44009	3.567897	0.659159
Li	-4.30781	5.760779	1.345924	Na	-4.15281	5.729235	1.247733
C	-7.38919	7.340103	5.340565	C	-7.35748	7.50521	5.572784
C	-7.08339	5.846678	5.483507	C	-6.85206	6.182655	6.157111
O	-6.06505	5.631542	4.483319	O	-6.23039	5.545698	5.02202
H	-7.9339	5.211745	5.244438	H	-7.64272	5.536103	6.523268
H	-6.67697	5.572867	6.451465	H	-6.09659	6.316909	6.928382
O	-6.84955	7.654524	4.039719	O	-6.61174	7.620205	4.343407
H	-6.87724	7.95408	6.078423	H	-7.13613	8.369146	6.190692
H	-8.44911	7.571611	5.337835	H	-8.41732	7.486886	5.327611
C	-6.03837	6.669184	3.639445	C	-6.05029	6.441851	4.042689
O	-5.36934	6.719335	2.645047	O	-5.46169	6.217491	3.024083
C	0.889001	5.774094	2.500704	C	1.400036	5.677976	1.918489
C	0.684829	6.714463	1.308987	C	1.165042	6.796222	2.937572
O	-0.75263	6.7984	1.206946	O	-0.27127	6.812654	3.069795
H	1.068662	6.31053	0.374483	H	1.485615	7.773043	2.581645
H	1.07614	7.713618	1.468635	H	1.597532	6.598044	3.912592
O	-0.40292	5.1472	2.641146	O	0.102598	5.523638	1.307109
H	1.114863	6.299007	3.426412	H	1.673994	4.731201	2.37925
H	1.628398	4.99982	2.324761	H	2.116347	5.933464	1.144701
C	-1.31362	5.824994	1.935234	C	-0.82546	6.129822	2.059456
O	-2.48815	5.58647	1.956621	O	-2.00407	6.069638	1.855407
C	-4.90205	9.064603	-2.75047	C	-6.06475	9.07465	-2.9054
C	-5.10227	9.83289	-1.44144	C	-5.00772	9.998087	-2.29293
O	-4.6821	8.879009	-0.44318	O	-4.29044	9.119077	-1.402
H	-6.14239	10.08797	-1.25046	H	-5.43581	10.80541	-1.7026
H	-4.48177	10.71816	-1.35307	H	-4.30656	10.40102	-3.01627
O	-4.82373	7.693007	-2.30802	O	-6.07949	7.954996	-1.99594
H	-3.9702	9.313071	-3.2542	H	-5.79138	8.708229	-3.89265
H	-5.73238	9.154753	-3.4431	H	-7.05955	9.506641	-2.93413
C	-4.62323	7.657975	-0.98597	C	-4.99594	7.996318	-1.2102
O	-4.41428	6.649586	-0.37054	O	-4.69464	7.135913	-0.43323
**$LiEMC_4$**				**$NaEMC_4$**			
Li	-4.49579	5.569992	1.190403	Na	-4.49198	5.595275	1.185827

Table S2. Countinued.

Atom	x	y	z	Atom	x	y	z
C	-2.83166	4.31466	-2.55569	C	-2.62262	4.285581	-2.69415
O	-1.68944	4.656294	-1.75192	O	-1.447	4.608046	-1.93332
H	-3.4511	5.191811	-2.72666	H	-3.23134	5.17385	-2.84636
H	-3.41831	3.537679	-2.07199	H	-3.20569	3.520047	-2.18834
H	-2.42164	3.952112	-3.49092	H	-2.25408	3.913834	-3.64295
C	-1.91933	5.12914	-0.53944	C	-1.62773	5.062074	-0.70417
O	-0.76886	5.385983	0.04731	O	-0.45203	5.31084	-0.16242
C	-0.79738	5.924112	1.397099	C	-0.42693	5.818703	1.197383
C	0.633738	6.109093	1.841411	C	1.019769	6.027467	1.575761
H	-1.33788	5.224046	2.032094	H	-0.91741	5.091354	1.842832
H	-1.34416	6.86585	1.376215	H	-0.99489	6.747414	1.22636
H	1.169223	5.161741	1.841151	H	1.574457	5.092199	1.531184
H	0.648325	6.510775	2.85418	H	1.076285	6.411187	2.593984
H	1.159737	6.80525	1.190809	H	1.497138	6.746101	0.912518
O	-3.02555	5.296762	-0.05443	O	-2.71018	5.221517	-0.16743
C	-7.86413	5.805068	2.459383	C	-8.27013	5.944235	2.241062
O	-8.33312	5.564552	1.121299	O	-8.69008	5.553116	0.923658
H	-7.14999	6.624285	2.470329	H	-7.62762	6.820276	2.190953
H	-7.40414	4.907279	2.864861	H	-7.74634	5.126235	2.730355
H	-8.74982	6.064277	3.027512	H	-9.18453	6.177801	2.773928
C	-7.43169	5.252175	0.20699	C	-7.74226	5.229836	0.058579
O	-8.0399	5.04213	-0.94189	O	-8.30299	4.888656	-1.08389
C	-7.22381	4.671157	-2.08557	C	-7.42881	4.496318	-2.17516
C	-8.14774	4.519971	-3.26969	C	-8.30357	4.177524	-3.36364
H	-6.70705	3.74252	-1.84756	H	-6.84587	3.63446	-1.85291
H	-6.47931	5.450285	-2.24144	H	-6.74541	5.319779	-2.378
H	-8.89402	3.74956	-3.08669	H	-8.98709	3.361432	-3.13788
H	-7.56831	4.233775	-4.14683	H	-7.67802	3.878098	-4.20388
H	-8.6606	5.4548	-3.48765	H	-8.88641	5.046216	-3.66357
O	-6.22938	5.169574	0.395931	O	-6.5451	5.246556	0.286146
C	-4.24703	8.72222	4.062039	C	-4.25207	8.946761	4.171486
O	-4.28968	9.405167	2.797907	O	-4.30519	9.696041	2.946646
H	-3.42823	8.007366	4.081063	H	-3.37121	8.309292	4.188245
H	-5.18806	8.210261	4.247669	H	-5.14799	8.341968	4.287369
H	-4.09065	9.50055	4.799518	H	-4.19434	9.690515	4.957562
C	-4.43223	8.664993	1.712152	C	-4.39699	9.007982	1.820543
O	-4.45172	9.450925	0.656214	O	-4.4283	9.848736	0.806036
C	-4.62674	8.838603	-0.65034	C	-4.53369	9.292695	-0.5313
C	-4.55216	9.941469	-1.67869	C	-4.53798	10.44854	-1.50281
H	-5.59213	8.334628	-0.66092	H	-5.45161	8.709312	-0.5871
H	-3.84535	8.093364	-0.78775	H	-3.68769	8.625864	-0.69239
H	-5.3322	10.68226	-1.51516	H	-5.37782	11.11408	-1.31367
H	-4.68525	9.518223	-2.67378	H	-4.62503	10.06727	-2.51952
H	-3.58615	10.4415	-1.64653	H	-3.61679	11.02329	-1.43067
O	-4.52887	7.449226	1.704976	O	-4.44492	7.792551	1.743768

Table S2. Countinued.

Atom	x	y	z	Atom	x	y	z
C	-4.50084	1.984696	1.47026	C	-4.61919	1.684287	1.732711
O	-4.25679	2.126046	2.880318	O	-4.35159	1.86127	3.13302
H	-3.65665	2.367103	0.901505	H	-3.79262	2.06551	1.137068
H	-5.40801	2.511701	1.186183	H	-5.54162	2.188731	1.454783
H	-4.61265	0.919185	1.308345	H	-4.71874	0.614062	1.594998
C	-4.12996	3.351266	3.358297	C	-4.21973	3.10267	3.571946
O	-3.90167	3.284079	4.653622	O	-3.97616	3.075057	4.866659
C	-3.74194	4.529179	5.384184	C	-3.81041	4.344708	5.551571
C	-3.49683	4.175498	6.831392	C	-3.50297	4.041873	6.998331
H	-4.64736	5.120095	5.25385	H	-4.73195	4.914345	5.43981
H	-2.90645	5.075417	4.948732	H	-3.004	4.892979	5.067271
H	-4.332	3.611868	7.242064	H	-4.31378	3.483218	7.461406
H	-3.38093	5.089121	7.413262	H	-3.37441	4.975776	7.544493
H	-2.59107	3.582402	6.941065	H	-2.58619	3.462741	7.089884
O	-4.21091	4.378988	2.706652	O	-4.31016	4.105943	2.886097
**$LiEC_5$**				**$NaEC_5$**			
C	-3.75324	-0.48338	1.945601	C	-5.44707	0.336081	0.522942
C	-2.67009	0.00741	0.979993	C	-4.19019	0.388241	-0.35036
O	-2.58558	1.41592	1.279356	O	-3.76436	1.757552	-0.22056
H	-2.94815	-0.10194	-0.06623	H	-4.39291	0.190316	-1.40127
H	-1.69612	-0.43882	1.152205	H	-3.38987	-0.25684	-0.00216
O	-4.40314	0.739038	2.349782	O	-5.79796	1.725153	0.665517
H	-3.3484	-0.96356	2.833975	H	-5.26041	-0.07443	1.513572
H	-4.49285	-1.12799	1.482445	H	-6.27916	-0.18431	0.059566
C	-3.64685	1.78797	2.00518	C	-4.76065	2.496837	0.297245
O	-3.89689	2.919398	2.312518	O	-4.72839	3.684556	0.413432
Li	-3.3688	4.783271	2.217327	C	-6.92292	7.565691	5.944236
C	-5.50818	6.485052	6.766536	C	-6.88082	6.035045	5.951041
C	-5.56151	4.954656	6.733822	O	-6.13809	5.728281	4.756132
O	-4.76994	4.630114	5.572343	H	-7.86669	5.581141	5.86796
H	-6.56558	4.564684	6.580726	H	-6.35338	5.6168	6.802455
H	-5.10944	4.482638	7.599901	O	-6.6035	7.890281	4.578266
O	-5.07136	6.820541	5.433662	H	-6.17003	8.015093	6.589213
H	-4.77643	6.872202	7.472544	H	-7.89969	7.977214	6.177459
H	-6.47193	6.952759	6.939594	C	-6.08027	6.814286	3.965705
C	-4.5912	5.728072	4.827495	O	-5.62441	6.825331	2.861887
O	-4.06378	5.740418	3.751183	C	0.550055	5.2219	3.08375
C	1.716967	6.18638	1.863608	C	0.723107	6.104001	1.843946
C	0.835454	7.223503	1.161774	O	-0.63027	6.467484	1.514995
O	-0.4981	6.807398	1.522024	H	1.150816	5.569125	0.997959
H	0.920027	7.193235	0.077596	H	1.288832	7.011448	2.030287
H	0.986459	8.237768	1.516481	O	-0.84127	4.858343	3.028675
O	0.790629	5.119528	2.152391	H	0.729445	5.75677	4.014571
H	2.130002	6.543216	2.80476	H	1.145636	4.314894	3.060233
H	2.511056	5.791739	1.238401	C	-1.4897	5.661964	2.166202

Table S2. Countinued.

Atom	x	y	z	Atom	x	y	z
C	-0.46471	5.561473	2.01106	O	-2.67196	5.659627	2.004818
O	-1.43452	4.915845	2.293082	C	-2.46443	9.23761	-3.20598
C	-5.7595	7.32324	-1.7623	C	-3.19842	10.26535	-2.33944
C	-6.67321	7.374574	-0.53461	O	-3.57061	9.500935	-1.17805
O	-5.87366	6.743166	0.496508	H	-4.10556	10.64476	-2.80609
H	-7.58718	6.800681	-0.65598	H	-2.57411	11.0943	-2.02174
H	-6.914	8.381284	-0.21657	O	-2.88332	7.98173	-2.6416
O	-4.71568	6.403279	-1.35719	H	-1.38138	9.307379	-3.12331
H	-5.29976	8.279486	-1.99353	H	-2.7554	9.260739	-4.25132
H	-6.24297	6.928438	-2.64688	C	-3.4446	8.187757	-1.4376
C	-4.80447	6.163152	-0.04939	O	-3.78506	7.316768	-0.69597
O	-4.00904	5.496822	0.560561	C	-9.51564	7.621726	-1.73264
C	-9.62602	10.91524	-4.96986	C	-10.183	6.607157	-0.80044
C	-10.7716	10.58767	-4.00841	O	-9.09958	6.191942	0.051559
O	-10.0962	9.949752	-2.90878	H	-10.5624	5.732316	-1.32522
H	-11.4891	9.883818	-4.42621	H	-10.966	7.033081	-0.18137
H	-11.291	11.46364	-3.63345	O	-8.11542	7.321436	-1.58624
O	-8.56737	10.0525	-4.51464	H	-9.68022	8.652924	-1.42531
H	-9.28716	11.94671	-4.89121	H	-9.78787	7.500637	-2.77633
H	-9.84421	10.67771	-6.00615	C	-7.92497	6.558394	-0.49321
C	-8.85487	9.58773	-3.28394	O	-6.85624	6.247395	-0.06509
O	-8.10767	8.940271	-2.61441	Na	-4.73443	5.946992	0.902976
**$LiEMC_5$**				**$NaEMC_5$**			
Li	-3.62527	6.44094	1.791736	Na	-4.7201	5.606256	1.426158
C	-2.92711	5.365141	-2.29948	C	-3.38987	5.517416	-2.83986
O	-1.65841	5.163032	-1.65297	O	-2.1373	5.515848	-2.1384
H	-3.25683	6.393653	-2.17432	H	-4.07262	6.243312	-2.40434
H	-3.67266	4.686563	-1.89266	H	-3.84093	4.528652	-2.8124
H	-2.74887	5.149851	-3.34652	H	-3.14587	5.791725	-3.85976
C	-1.60899	5.354599	-0.34588	C	-2.16778	5.195533	-0.85065
O	-0.38459	5.118689	0.077268	O	-0.93447	5.203026	-0.38508
C	-0.1178	5.251826	1.499766	C	-0.7329	4.845564	1.010306
C	1.356495	5.001268	1.707132	C	0.744211	4.968485	1.297846
H	-0.73362	4.526083	2.029071	H	-1.08698	3.825355	1.151737
H	-0.41484	6.251005	1.812531	H	-1.33146	5.51481	1.624178
H	1.633926	4.002237	1.376759	H	1.324973	4.30514	0.659863
H	1.59325	5.088156	2.766972	H	0.933336	4.69623	2.33578
H	1.957091	5.726482	1.161672	H	1.090291	5.988758	1.143146
O	-2.55482	5.693421	0.344626	O	-3.17582	4.938987	-0.21833
C	-9.80191	4.184519	1.460921	C	-8.12069	3.838783	2.783686
O	-9.88694	3.165699	0.4538	O	-7.9988	2.927089	1.678531
H	-9.46158	5.124011	1.03198	H	-8.55644	4.77761	2.450638
H	-9.12543	3.876568	2.255428	H	-7.1486	4.017192	3.236315
H	-10.8096	4.289173	1.846903	H	-8.78446	3.346373	3.485179
C	-8.76133	2.870191	-0.19032	C	-7.21486	3.297926	0.67717

Table S2. Countinued.

Atom	x	y	z	Atom	x	y	z
O	-9.02441	1.909104	-1.06606	O	-7.25476	2.365425	-0.25941
C	-7.92175	1.433702	-1.87406	C	-6.49267	2.601248	-1.47188
C	-8.45494	0.343199	-2.77326	C	-6.66321	1.389561	-2.3567
H	-7.13845	1.068851	-1.21085	H	-5.45247	2.772312	-1.19901
H	-7.52327	2.272356	-2.44392	H	-6.87563	3.505443	-1.94329
H	-8.85442	-0.48346	-2.18876	H	-6.28736	0.491614	-1.86977
H	-7.64951	-0.03756	-3.40054	H	-6.10765	1.534671	-3.28265
H	-9.24427	0.722111	-3.42006	H	-7.71052	1.234057	-2.60777
O	-7.68333	3.393099	-0.0062	O	-6.56512	4.324845	0.630716
C	-1.63362	8.866861	4.756849	C	-3.04311	8.088216	4.707089
O	-1.79772	9.724853	3.615022	O	-2.99538	8.994952	3.596463
H	-0.92958	8.069283	4.531899	H	-2.17955	7.427256	4.693549
H	-2.58919	8.442779	5.054407	H	-3.95691	7.499439	4.680064
H	-1.24094	9.506345	5.538594	H	-3.02148	8.715569	5.590679
C	-2.31847	9.187672	2.524554	C	-3.02455	8.455964	2.382529
O	-2.38741	10.10717	1.584288	O	-2.9189	9.425205	1.492399
C	-2.90866	9.716226	0.285118	C	-2.89754	9.051812	0.090696
C	-2.92583	10.95065	-0.58416	C	-2.75448	10.32159	-0.71395
H	-3.90291	9.296909	0.425951	H	-3.82356	8.525171	-0.13416
H	-2.2601	8.940844	-0.11994	H	-2.0609	8.373608	-0.06997
H	-3.57248	11.71759	-0.16212	H	-3.59062	10.99529	-0.53588
H	-3.30268	10.69153	-1.57306	H	-2.72957	10.07744	-1.77546
H	-1.92532	11.36354	-0.69703	H	-1.83172	10.84071	-0.46253
O	-2.67277	8.026078	2.421399	O	-3.12809	7.268594	2.144865
C	-4.7254	2.979929	1.712363	C	-3.76701	1.757872	1.746445
O	-4.36395	2.900024	3.107108	O	-3.44358	1.95417	3.13296
H	-3.92641	3.442541	1.14026	H	-3.44742	2.607424	1.14918
H	-5.65022	3.535557	1.589075	H	-4.83769	1.602605	1.633684
H	-4.87108	1.952135	1.402542	H	-3.23286	0.860698	1.454702
C	-3.96329	4.004287	3.704192	C	-3.84124	3.083606	3.699813
O	-3.71629	3.741957	4.973669	O	-3.53359	3.039636	4.983226
C	-3.25992	4.832888	5.814714	C	-3.88807	4.182945	5.801377
C	-3.03223	4.273242	7.198426	C	-3.43943	3.886734	7.212421
H	-4.0223	5.610658	5.809033	H	-4.96539	4.32987	5.738065
H	-2.34818	5.241749	5.381907	H	-3.39546	5.062387	5.390012
H	-3.94948	3.851457	7.604869	H	-3.93253	2.997304	7.599643
H	-2.69891	5.070627	7.861952	H	-3.69318	4.726643	7.858254
H	-2.2698	3.496704	7.185964	H	-2.36294	3.732544	7.256689
O	-3.83569	5.096924	3.176594	O	-4.39584	4.006289	3.131635
C	-6.88928	6.601402	-0.98125	C	-7.51633	7.370721	-0.97535
O	-7.42734	7.109713	0.255889	O	-7.94968	8.14661	0.153789
H	-6.67081	5.542426	-0.87506	H	-7.33196	6.340738	-0.68387
H	-5.99824	7.156455	-1.26113	H	-6.61475	7.805659	-1.40252
H	-7.67502	6.752448	-1.71261	H	-8.32942	7.430702	-1.68973
C	-6.59875	7.223332	1.276258	C	-7.17836	8.129823	1.231032

Table S2. Countinued.

Atom	x	y	z	Atom	x	y	z
O	-7.2714	7.64067	2.332211	O	-7.71692	8.91444	2.146564
C	-6.5303	7.879558	3.557929	C	-7.00984	9.070552	3.40375
C	-7.52636	8.294133	4.614155	C	-7.82269	10.00623	4.266113
H	-5.795	8.659293	3.363723	H	-6.01893	9.469635	3.193255
H	-6.00233	6.965741	3.82422	H	-6.89921	8.087081	3.85944
H	-8.05479	9.198664	4.31983	H	-7.93608	10.9767	3.787185
H	-7.00297	8.49369	5.548588	H	-7.31648	10.15339	5.219339
H	-8.25747	7.508022	4.792872	H	-8.81257	9.598671	4.462128
O	-5.40302	6.989935	1.23943	O	-6.14575	7.497266	1.352646
**$LiClO_4$**				**$NaClO_4$**			
Cl	0.161649	-0.94633	0.013712	Cl	0.179816	-1.24035	0.177823
O	-1.16156	-1.461	0.06654	O	-1.30391	-1.24437	0.107766
O	0.588914	-0.38567	1.247269	O	0.607712	-0.4873	1.312008
O	1.113534	-2.01052	-0.42807	O	0.666314	-2.58178	0.198444
O	0.281044	0.062767	-1.08232	O	0.620052	-0.56461	-1.0695
Li	1.523516	-1.01654	-1.94104	Na	-1.42479	-0.20628	-1.84564
**LiOTf**				**NaOTf**			
S	0.340603	-0.00391	-0.26612	S	0.249942	0.004687	-0.25325
O	1.779354	-0.06926	-0.59843	O	1.679473	0.086704	-0.59233
O	-0.2075	1.269605	0.055621	O	-0.40879	1.228358	0.075859
C	0.199847	-1.02899	1.283675	C	0.248707	-1.00209	1.314654
F	-1.07485	-1.15123	1.647286	F	-0.99533	-1.24659	1.726235
F	0.883697	-0.46642	2.277612	F	0.906988	-0.37277	2.288145
F	0.699857	-2.25753	1.064393	F	0.857577	-2.18749	1.096867
O	-0.35615	-0.81529	-1.28617	O	-0.42632	-0.88053	-1.21448
Li	1.326931	-1.21757	-2.01206	Na	1.479546	-1.37087	-2.2759
**$LiPF_6$**				**$NaPF_6$**			
P	0.788525	-1.08728	0.054082	P	0.685927	-0.79447	0.17413
F	-0.89709	-1.21719	-0.02178	F	-0.92631	-0.55467	0.570245
F	0.492553	0.553055	-0.23504	F	0.933875	0.767532	0.216745
F	0.713603	-0.84189	1.605554	F	1.037103	-0.99444	1.70381
F	2.338506	-0.83892	-0.03871	F	2.157077	-1.11719	-0.31045
F	0.917579	-2.64936	0.179187	F	0.283085	-2.41803	0.048995
F	0.692169	-1.21439	-1.6297	F	0.18092	-0.676	-1.42122
Li	-0.77664	-0.04593	-1.4876	Na	-1.60337	-1.94423	-1.02986
**LiTFSI**				**NaTFSI**			
C	-0.55581	1.200973	-1.69475	C	-0.54827	1.20386	-1.67609
S	1.111305	0.796028	-0.95361	S	1.130595	0.74204	-1.00036
O	1.328654	1.910241	-0.02242	O	1.433558	1.854539	-0.10505
O	2.01569	0.685568	-2.04502	O	1.973104	0.573736	-2.13653
N	0.84227	-0.58023	-0.25386	N	0.836534	-0.62363	-0.28896
F	-0.4763	2.365381	-2.33602	F	-0.45458	2.358181	-2.33857
F	-0.9321	0.255512	-2.5436	F	-1.01054	0.268483	-2.49358
F	-1.46819	1.30711	-0.72121	F	-1.41608	1.365753	-0.66477
S	0.702449	-0.96024	1.260025	S	0.669726	-1.01294	1.220268

Table S2. Countinued.

Atom	x	y	z	Atom	x	y	z
O	-0.32954	-1.91068	1.489883	O	-0.3151	-2.02683	1.396553
O	0.764006	0.186747	2.174542	O	0.642629	0.111391	2.150952
C	2.313019	-1.84381	1.603358	C	2.308342	-1.82344	1.603138
F	2.334942	-2.23068	2.877446	F	2.312683	-2.23137	2.873453
F	3.338637	-1.01086	1.388764	F	3.302668	-0.93619	1.440451
F	2.441198	-2.90405	0.81886	F	2.526295	-2.86331	0.810683
Li	1.151673	1.891691	1.764605	Na	1.190332	2.198435	2.015417

Table S3. Calculated properties of the $[cation]_1[anion]_1$ and $[cation]_1[anion]_x$ ion pairs.

Sample	ΔE_d[a] (kJ mol^{-1})	Sample	ΔE[b] (kJ mol^{-1})
$[Li]_1[TFSI]_1$	591	$[Li]_1[EC]_1$	219
$[Na]_1[TFSI]_1$	482	$[Li]_1[EMC]_1$	200
$[Li]_1[PF_6]_1$	581	$[Li]_1[EC]_4$	534
$[Na]_1[PF_6]_1$	493	$[Li]_1[EC]_2[EMC]_2$	510
$[Li]_1[ClO_4]_1$	602	$[Li]_1[EMC]_4$	466
$[Na]_1[ClO_4]_1$	506	$[Na]_1[EC]_1$	157
$[Li]_1[OTf]_1$	599	$[Na]_1[EMC]_1$	134
$[Na]_1[OTf]_1$	505	$[Na]_1[EC]_4$	427
		$[Na]_1[EC]_2[EMC]_2$	403
		$[Na]_1[EMC]_4$	363

[a] Dissociation energy. [b] Stabilization energy.

Table S4. Assignments of the Raman band for the species reported in this work.

Sample	Raman band (cm^{-1})	
	Free	Bound
EC (C–O)	895[17]	905 (Li)[17] / 900 (Na)[18]
EMC (C–O)	918[17] / 930[19]	935 (Li)[17] / 945 (Li)[19] / 938 (Na)[19]
$TFSI^-$	742[20]	745–750 (Li)[20a] / 745 (Na)[20b]
PF_6^-	742[20a]	746 (Li)[20a] / 743 (Na)[18]
ClO_4^-	935[21]	940–950 (Li)[21] / 940–945 (Na)[22]
OTf^-	757[23]	760–763 (Li)[23]

Table S5. Coordination number calculated from the peak fitting data of the Raman spectra.

Sample[a]	$[EC]/[M^+]$	$[EMC]/[M^+]$	Coord. EC	Coord. EMC	CN	[EC]/[EMC]	Coord. anion	Free anion
LiTFSI	8.34	12.59	1.84	1.77	3.61	1.04	21.8%	78.2
$LiPF_6$	8.78	13.26	1.80	1.75	3.55	1.02	22.8%	77.2
$LiClO_4$	9.00	13.60	1.18	N/A	N/A	N/A	N/A	N/A
LiOTf	8.71	13.17	0.70	1.34	1.35	1.07	60.7	39.3
NaTFSI	8.55	12.91	1.73	2.16	3.88	0.80	24.5	75.5
$NaPF_6$	8.85	13.37	1.76	2.05	3.81	0.86	25.0	75.0
$NaClO_4$	9.01	13.61	1.13	N/A	N/A	N/A	N/A	N/A
NaOTf	8.92	13.47	0.96	1.06	2.03	0.91	29.4	70.6
EC-EMC	N/A	N/A	N/A	N/A	N/A	0.66	N/A	N/A

[a] The electrolytes are abbreviated in the identity of conducting salt.

Table S6. Crystallographic information of lithium and sodium-based solvates.

Sample	Coordination	Type
**Salt**		
$LiPF_6$	6-fold Li–F coordination[24]	Salt
$LiAsF_6$	6-fold Li–F coordination[25]	Salt
LiTFSI	4-fold Li–O coordination[26]	Salt
$LiClO_4$	6-fold Li–O coordination[27]	Salt
LiOTf	4-fold Li–O coordination[25]	Salt
**Solvates**		
$[LiOTf][EC]_1$	1-fold Li–O (solvent) + 3-fold Li–O (anion) coordination[28]	AGG
$[LiOTf][G1]_{0.5}$	1-fold Li–O (solvent) + 3-fold Li–O (anion) coordination[29]	AGG
$[LiOTf][G2]_1$	3-fold Li–O (solvent) + 2-fold Li–O (anion) coordination[30]	AGG
$[LiClO_4][EC]_1$	2-fold Li–O (solvent) + 2-fold Li–O (anion) coordination[28]	AGG
$[LiTFSI][G2]_{0.5}$	3-fold Li–O (solvent) + 2-fold Li–O (anion) coordination[31]	AGG
$[LiAsF_6][G3]_1$	4-fold Li–O (solvent) + 1-fold Li–F (anion) coordination[32]	CIP
$[LiOTf][G3]_1$	4-fold Li–O (solvent) + 2-fold Li–O (anion) coordination[32]	CIP
$[LiClO_4][EC]_3$	3-fold Li–O (solvent) + 1-fold Li–O (anion) coordination[28]	CIP
$[LiClO_4][G3]_1$	4-fold Li–O (solvent) + 1-fold Li–O (anion) coordination[32]	CIP
$[LiClO_4][G1]_2$	4-fold Li–O (solvent) + 2-fold Li–O (anion) coordination[33]	CIP
$[LiPF_6][GBL]_4$	5-fold Li–O (solvent) coordination[28]	SSIP
$[LiPF_6][EC]_4$	4-fold Li–O (solvent) coordination[28]	SSIP
$[LiAsF_6]P[EO]_6$	5-fold Li–O (solvent) coordination[34]	SSIP
$[LiClO_4][G2]_2$	6-fold Li–O (solvent) coordination[33]	SSIP
$[LiTFSI][G2]_2$	6-fold Li–O (solvent) coordination[31]	SSIP
**Salt**		
$NaPF_6$	6-fold Na–F coordination[35]	Salt
NaTFSI	6-fold Na–O coordination[36]	Salt
$NaClO_4$	8-fold Na–O coordination[37]	Salt
NaOTf	N/A	Salt
**Solvates**		
$[NaOTf]P[EO]_1$	2-fold Na–O (solvent) + 4-fold Na–O (anion) coordination[38]	AGG
$[NaClO_4]P[EO]_1$	3-fold Na–O (solvent) + 2-fold Na–O (anion) coordination[39]	AGG
$[NaTFSI][G4]_1$	5-fold Na–O (solvent) + 2-fold Na–O (anion) coordination[40]	AGG
$[NaPF_6][G5]_1$	6-fold Na–O (solvent) + 1-fold Na–F (anion) coordination[40]	CIP
$[NaClO_4][G4]_1$	5-fold Na–O (solvent) + 2-fold Na–O (anion) coordination[40]	CIP
$[NaTFSI][G5]_1$	6-fold Na–O (solvent) + 1-fold Na–O (anion) coordination[40]	CIP
$[NaClO_4][G5]_1$	6-fold Na–O (solvent) + 1-fold Na–O (anion) coordination[40]	CIP

4. Supplementary Figures

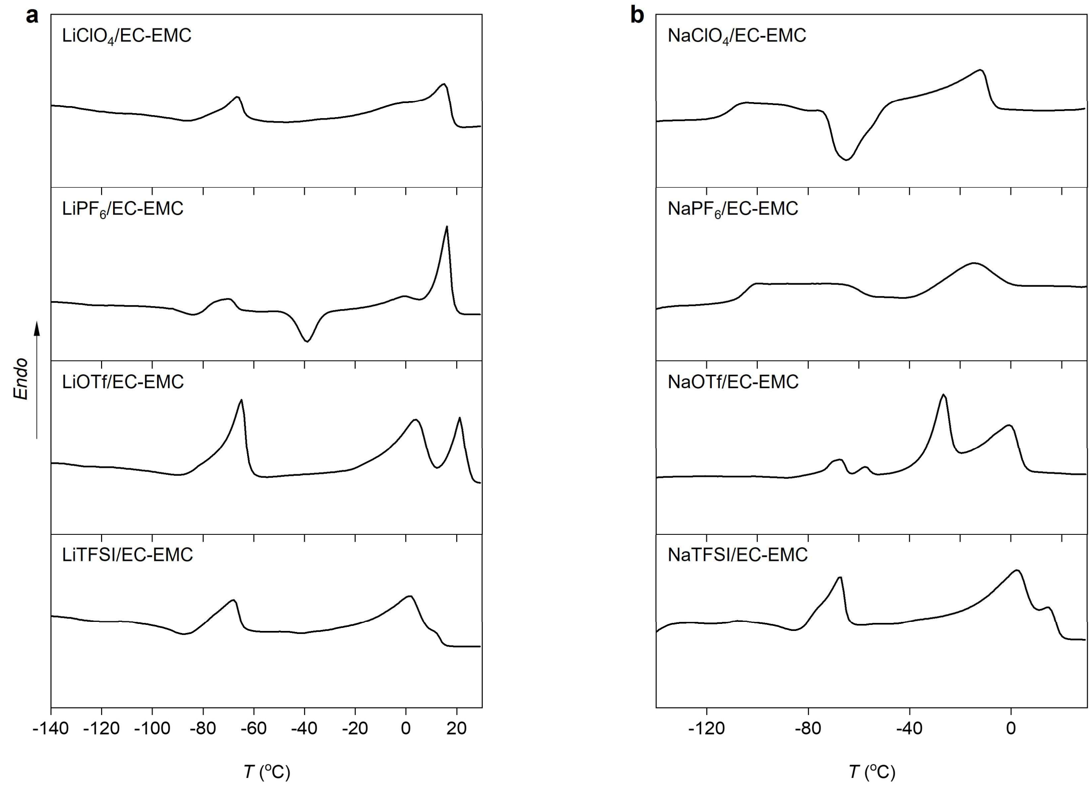

Figure S1. DSC traces of the lithium- (a) and sodium- (b) electrolytes.

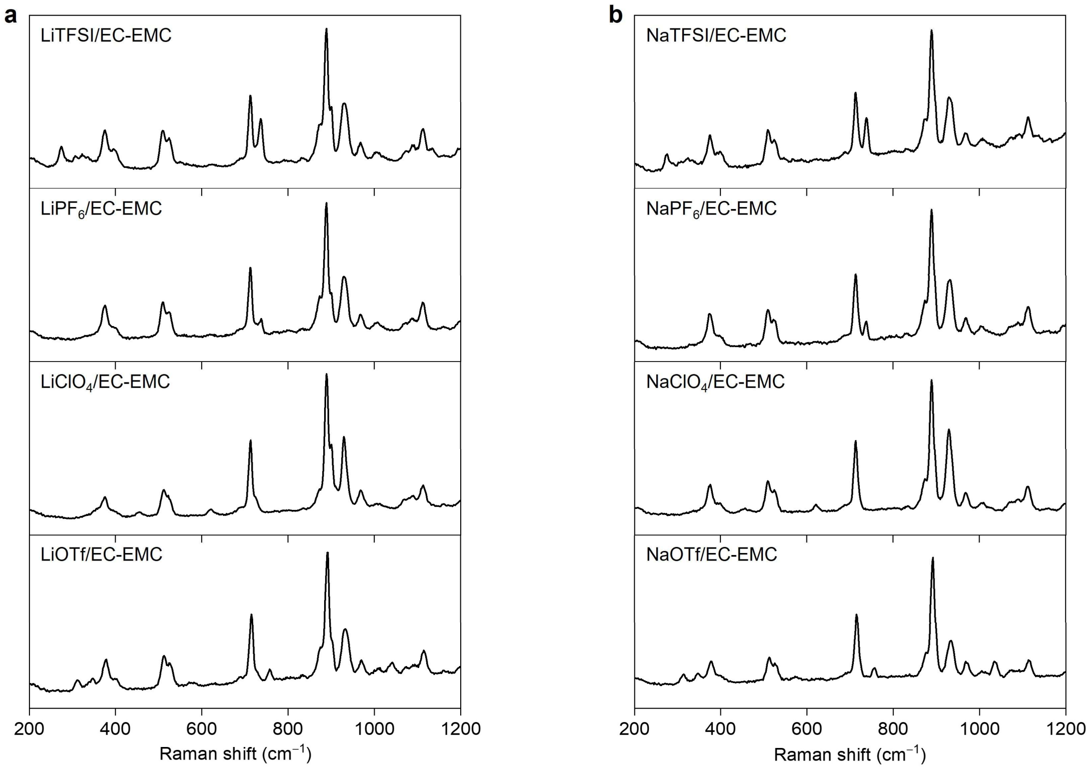

Figure S2. Raman spectra of the lithium- (a) and sodium-based (b) electrolytes.

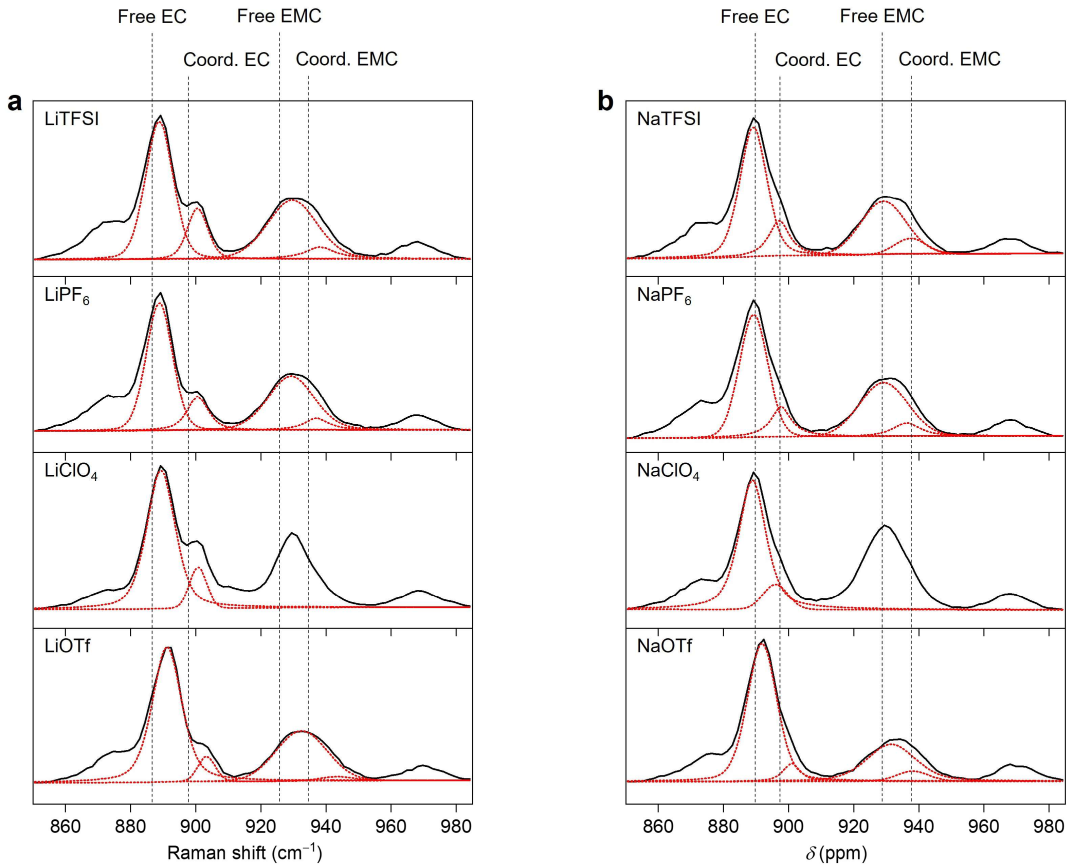

Figure S3. Zoomed-in Raman spectra (850–985 cm^{-1}) of the lithium- (a) and sodium-based (b) electrolytes.

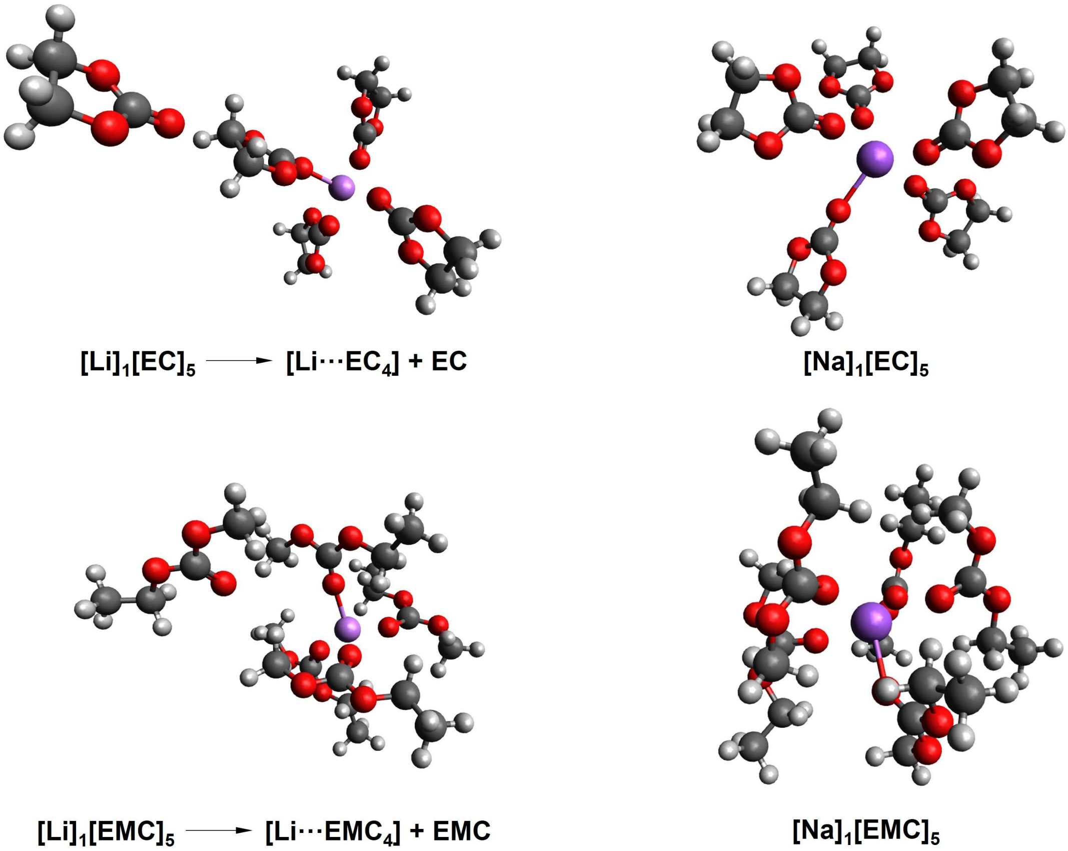

Figure S4. Optimized atomic structures of representative [cation]/[solvent] pairs.

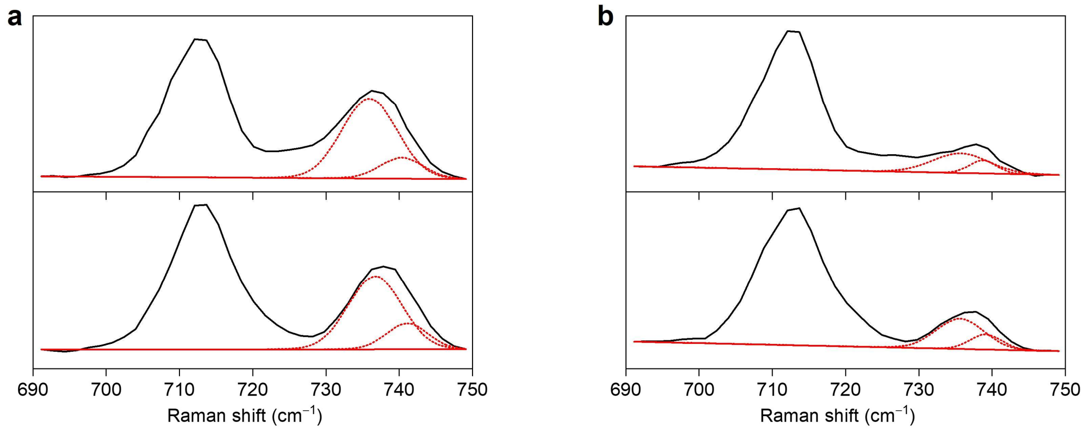

Figure S5. Zoomed-in Raman spectra (690–750 cm^{-1}) of the TFSI- (a) and PF_6-based (b) electrolytes.

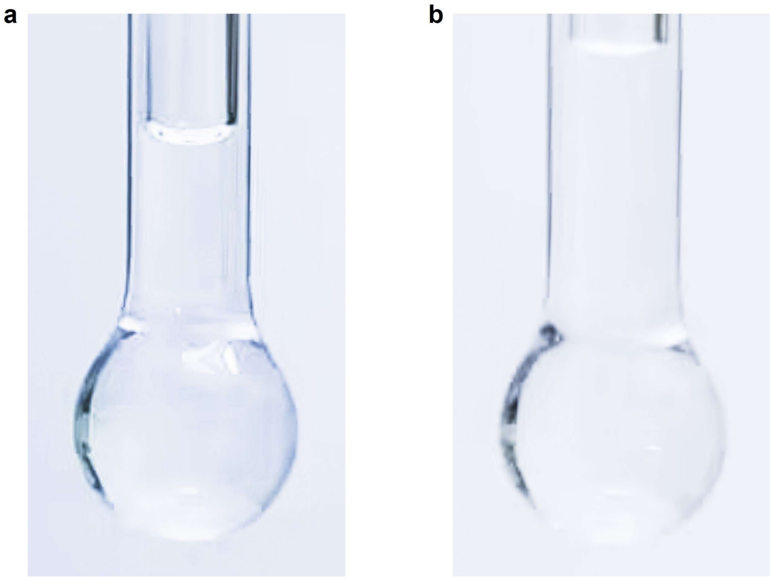

Figure S6. Digital image of the $LiPF_6$ (a) and $NaPF_6$ (b) electrolytes before thermal storage tests.

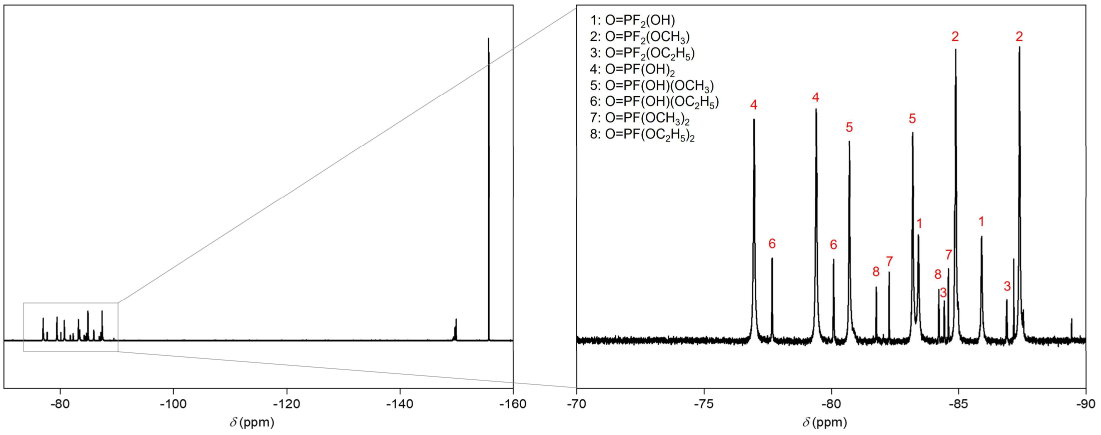

Figure S7. ^{19}F NMR spectrum of $LiPF_6$-based electrolytes after thermal storage at 80 °C for 14 days.

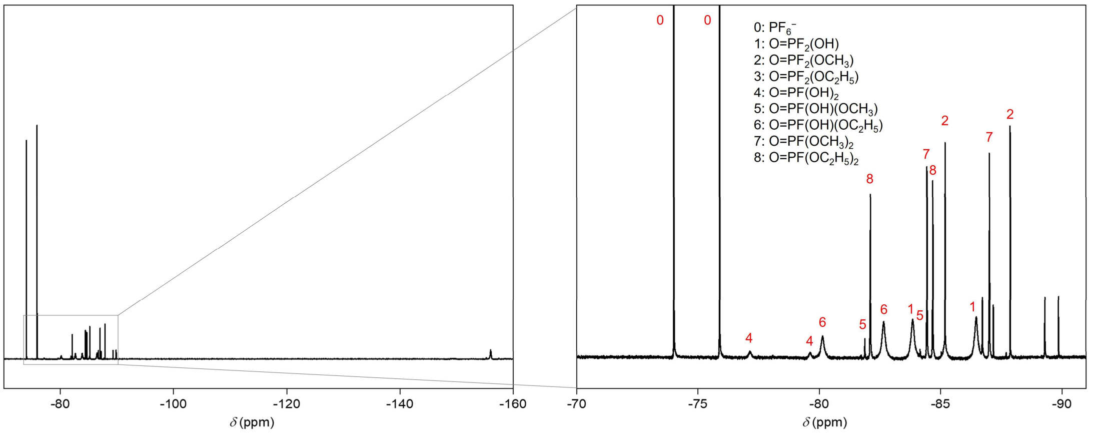

Figure S8. ^{19}F NMR spectrum of $NaPF_6$-based electrolytes after thermal storage at 80 °C for 14 days.

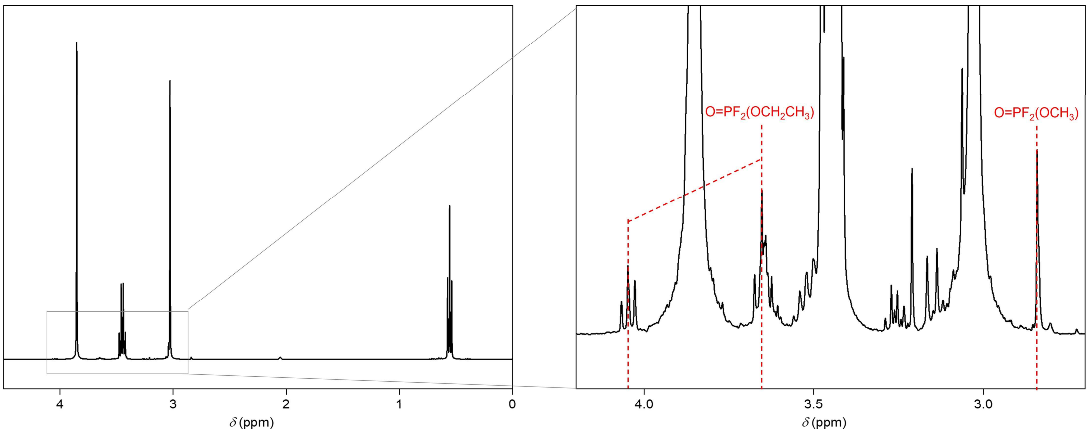

Figure S9. ^{1}H NMR spectrum of $LiPF_6$-based electrolytes after thermal storage at 80 °C for 14 days.

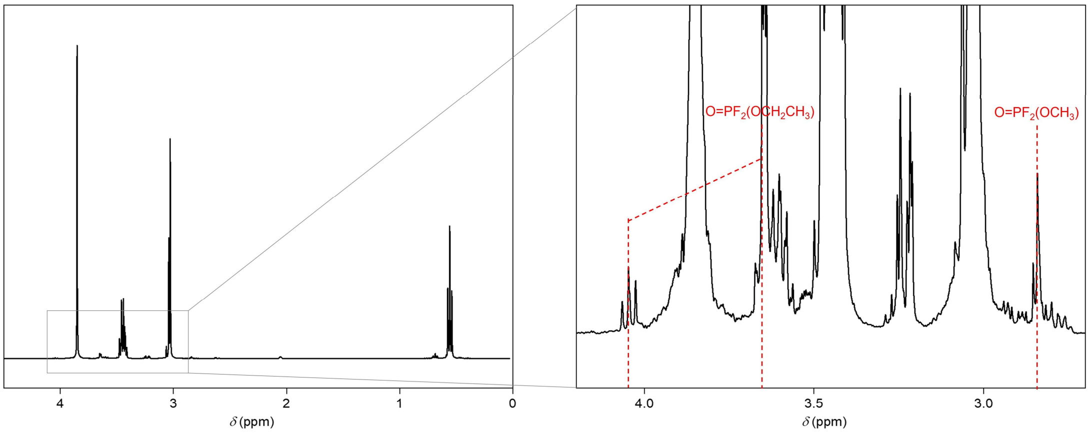

Figure S10. ^{1}H NMR spectrum of $NaPF_6$-based electrolytes after thermal storage at 80 °C for 14 days.

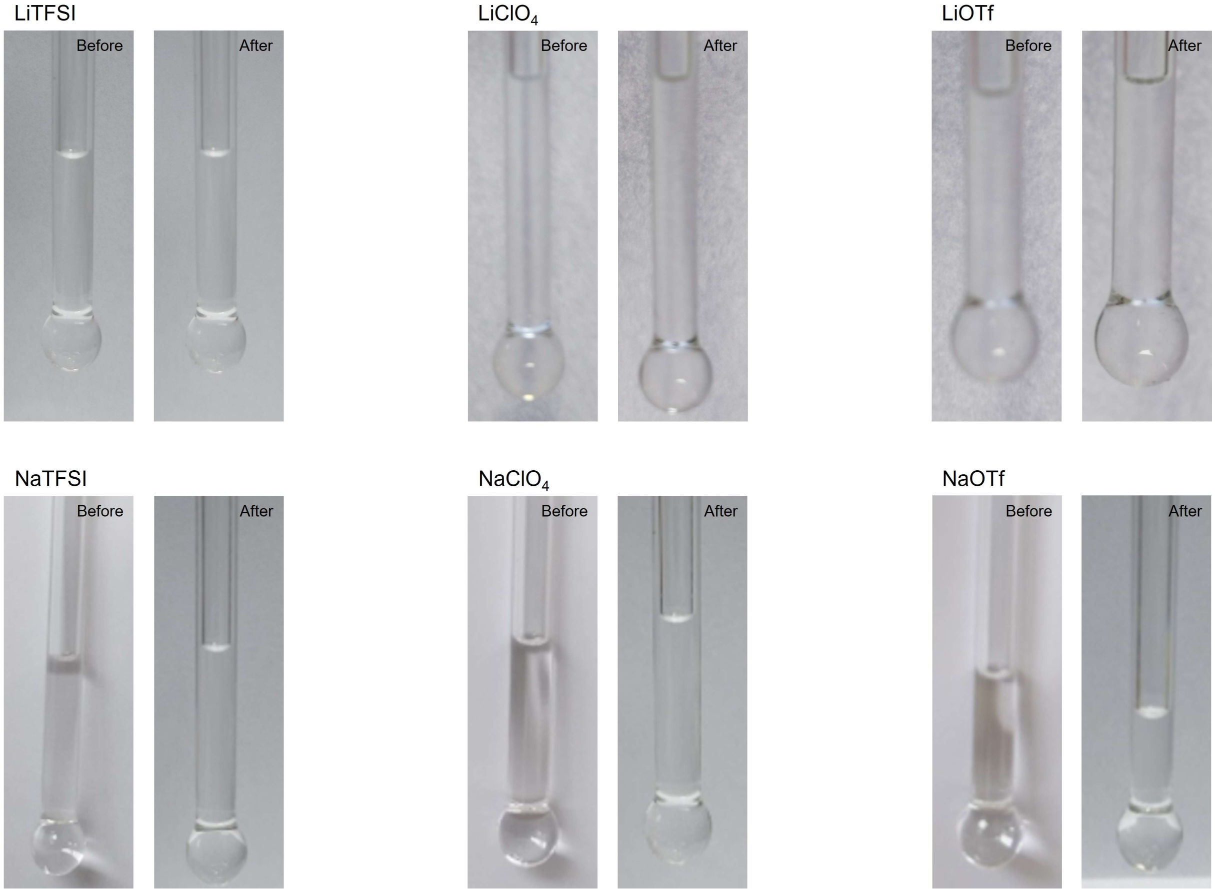

Figure S11. Optical images of the lithium- and sodium-based electrolytes before and after thermal storage at 80 °C for 14 days.

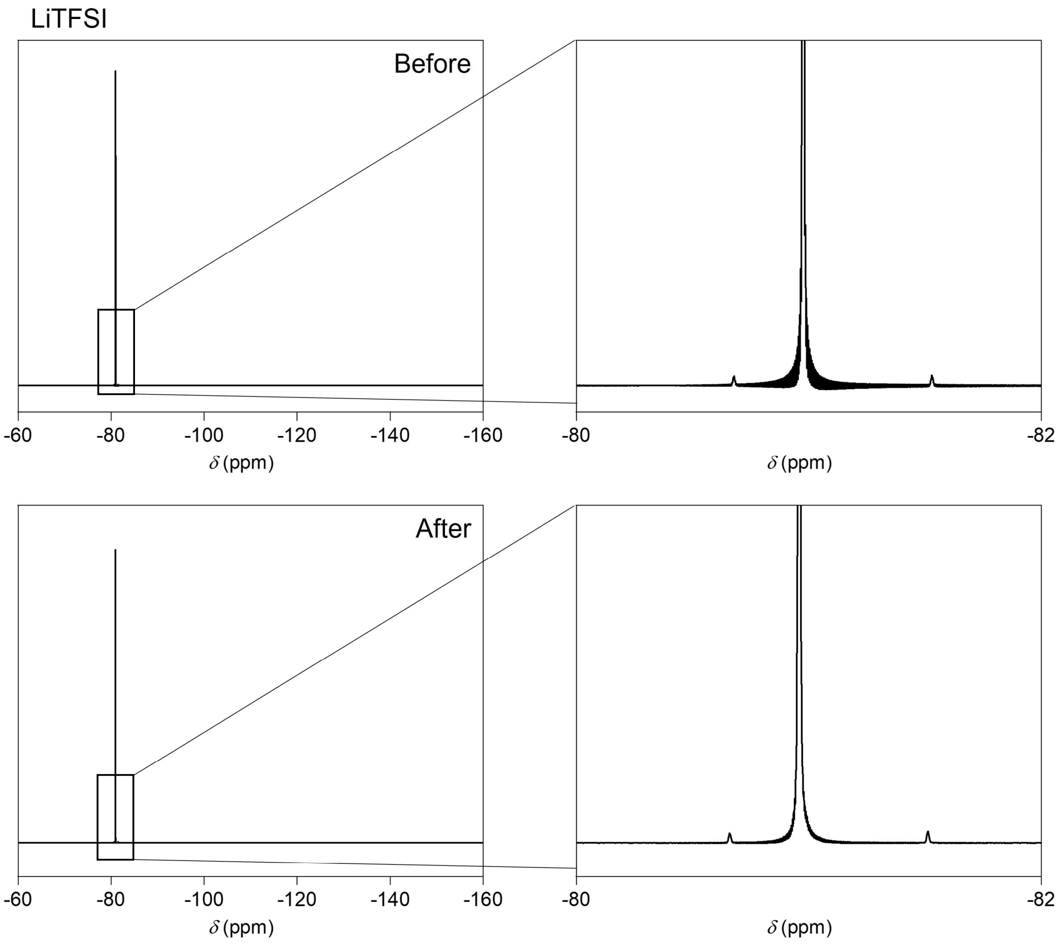

Figure S12. ^{19}F NMR spectra of LiTFSI-based electrolytes before (upper) and after (bottom) after thermal storage at 80 °C for 14 days.

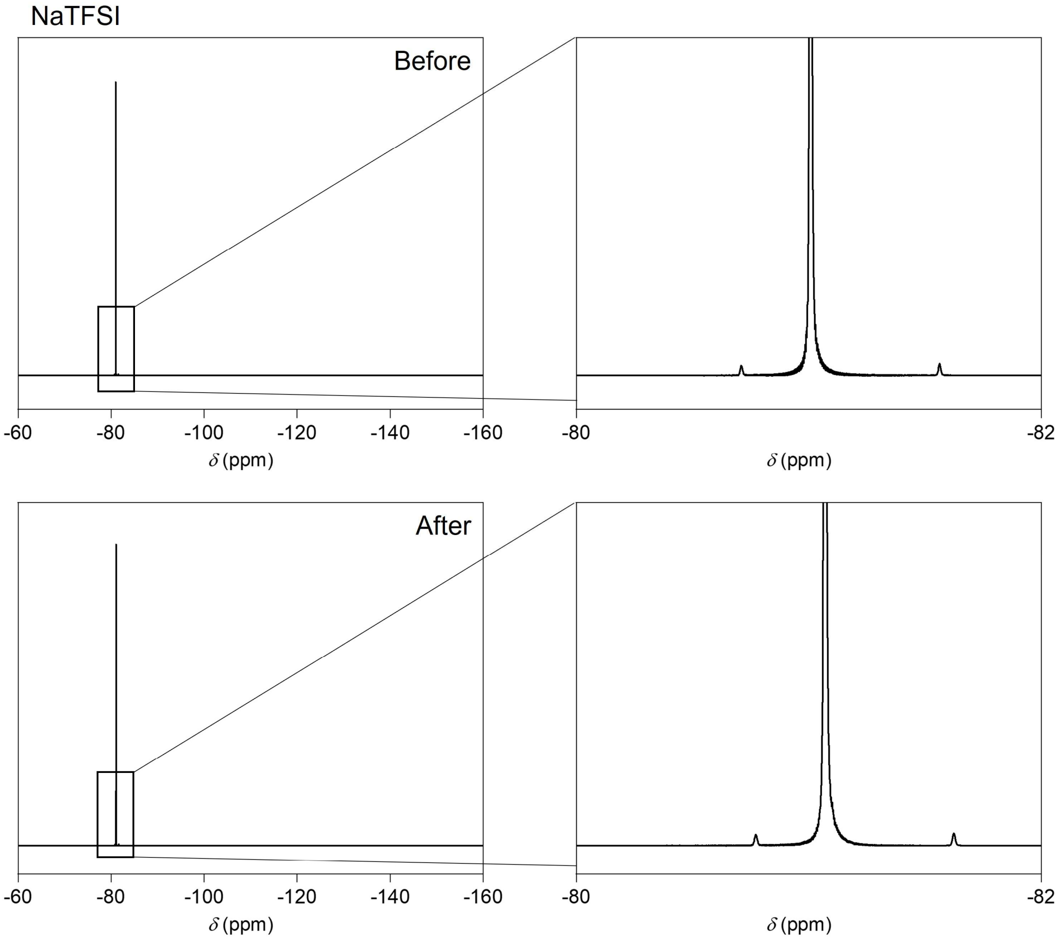

Figure S13. ^{19}F NMR spectra of NaTFSI-based electrolytes before (upper) and after (bottom) after thermal storage at 80 °C for 14 days.

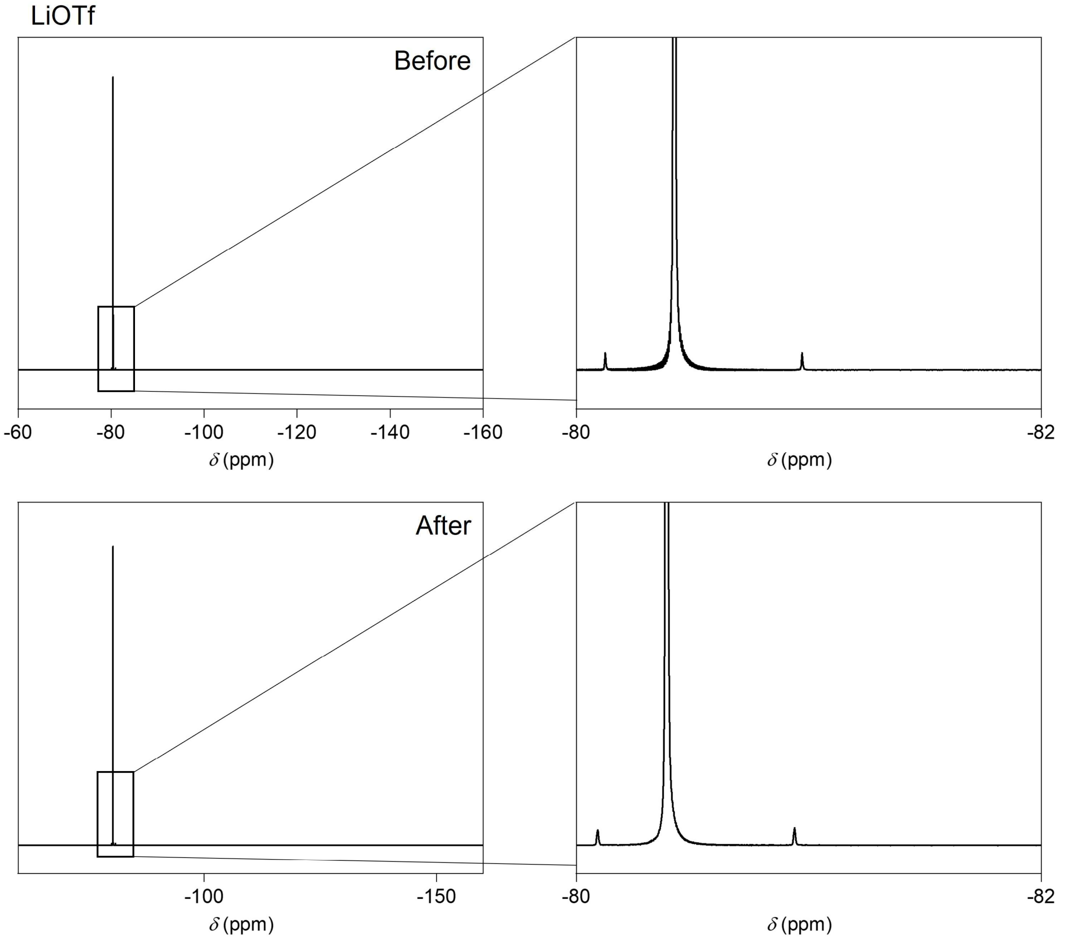

Figure S14. ^{19}F NMR spectra of LiOTf-based electrolytes before (upper) and after (bottom) after thermal storage at 80 °C for 14 days.

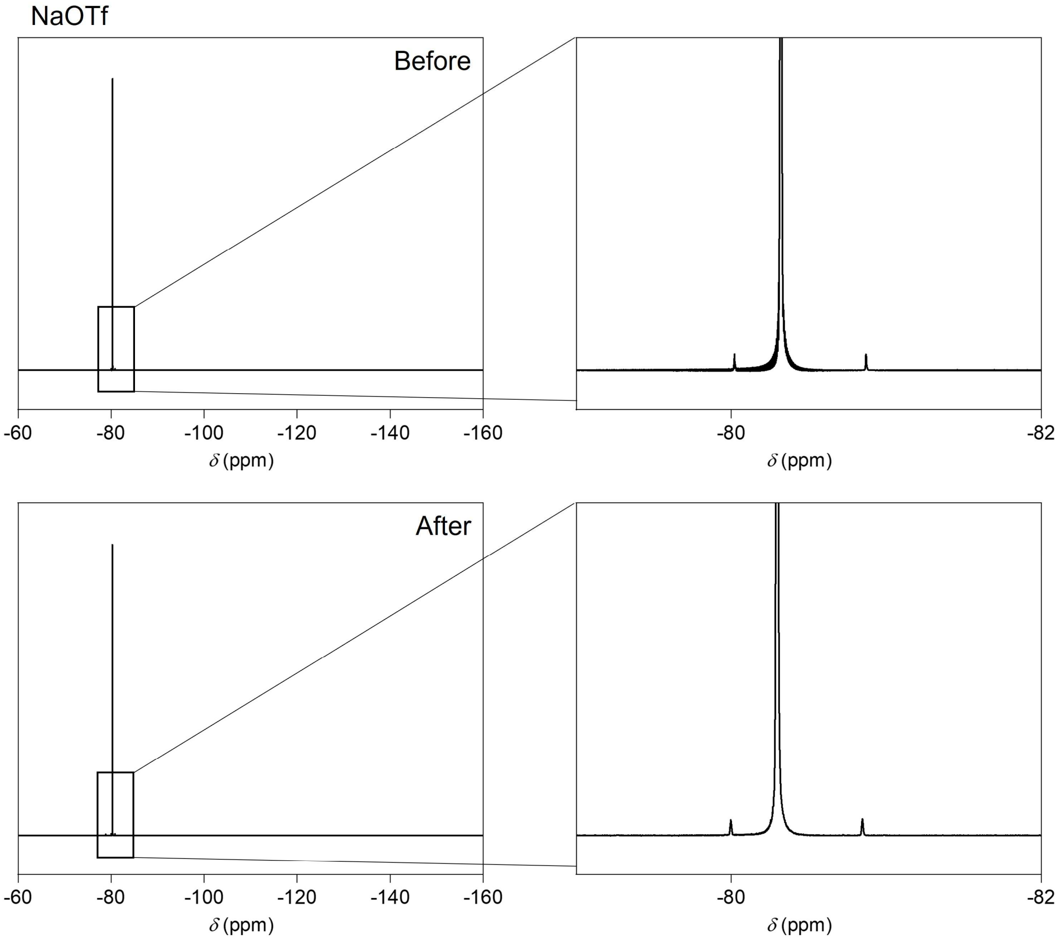

Figure S15. ^{19}F NMR spectra of NaOTf-based electrolytes before (upper) and after (bottom) after thermal storage at 80 °C for 14 days..

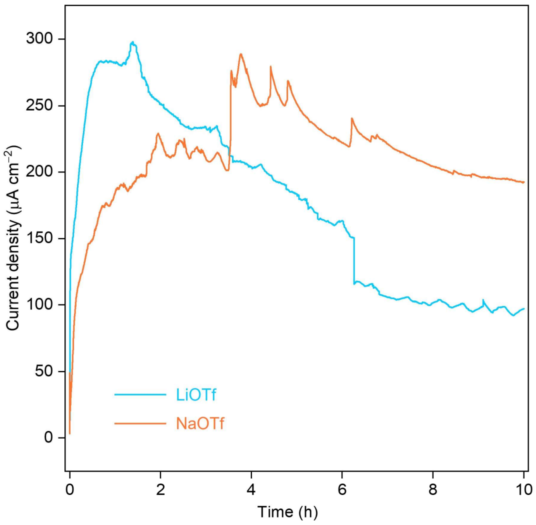

Figure S16. Chronoamperometry curves of aluminum foils recovered from OTf-based samples after DC polarization measurements

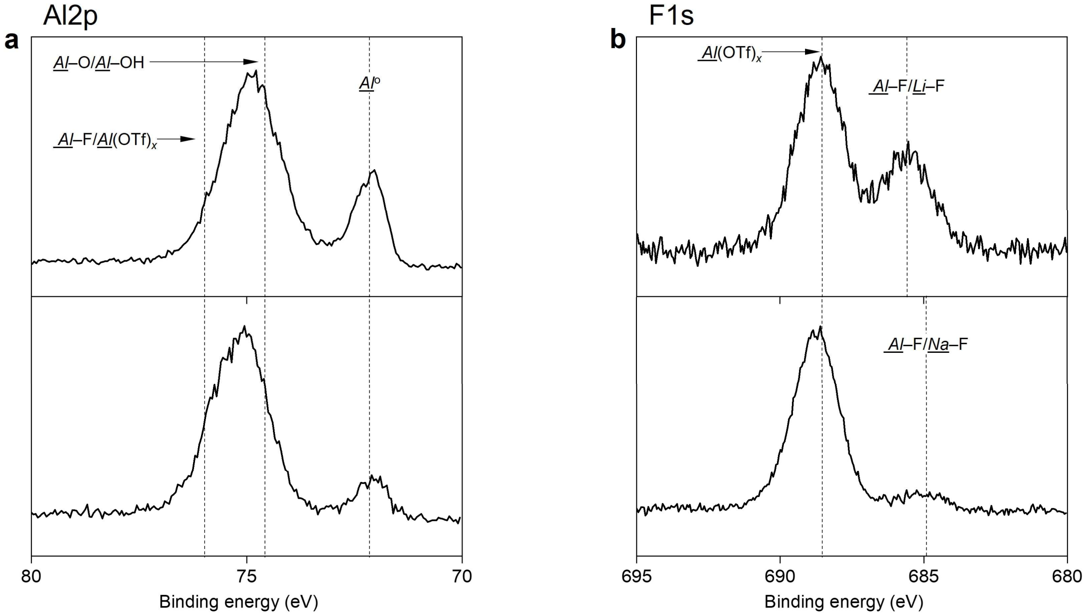

Figure S17. Al2p (a) and F1s (b) XPS spectra of aluminum foils recovered from LiOTf- or NaOTf-based electrolytes.

References

[1] a) H. Zhang, F. Chen, O. Lakuntza, U. Oteo, L. Qiao, M. Martinez-Ibanez, H. Zhu, J. Carrasco, M. Forsyth, M. Armand, *Angew. Chem. Int. Ed.* **2019**, 58, 12070; b) V. Havu, V. Blum, P. Havu, M. Scheffler, *J. Comput. Phys.* **2009**, 228, 8367.

[2] O. Borodin, G. D. Smith, R. L. Jaffe, *J. Comput. Chem.* **2001**, 22, 641.

[3] H. Zhang, O. Arcelus, J. Carrasco, *Electrochim. Acta* **2018**, 280, 290.

[4] W. L. F. Armarego, C. L. L. Chai, in *Purification of Laboratory Chemicals (Sixth Edition)*, (Eds: W. L. F. Armarego, C. L. L. Chai), Butterworth-Heinemann, Oxford 2009.

[5] L. Zheng, H. Zhang, P. Cheng, Q. Ma, J. Liu, J. Nie, W. Feng, Z. Zhou, *Electrochim. Acta* **2016**, 196, 169.

[6] Z. Song, X. Wang, W. Feng, M. Armand, Z. Zhou, H. Zhang, *Adv. Mater.* **2024**, 36, 2310245.

[7] W. A. Henderson, N. R. Brooks, W. W. Brennessel, V. G. Young, *J. Phys. Chem. A* **2004**, 108, 225.

[8] a) W. A. Henderson, *J. Phys. Chem. B* **2006**, 110, 13177; b) W. A. Henderson, F. McKenna, M. A. Khan, N. R. Brooks, V. G. Young, Jr., R. Frech, *Chem. Mater.* **2005**, 17, 2284.

[9] W. A. Henderson, *J. Phys. Chem. B* **2006**, 110, 13177.

[10] J. Chidiac, L. Timperman, M. Anouti, *J. Taiwan Inst. Chem. Eng.* **2021**, 126, 88.

[11] a) H. Wu, Z. Song, X. Wang, W. Feng, Z. Zhou, H. Zhang, *Nano Res.* **2023**, 16, 8269; b) Z. Y. Song, L. P. Zheng, P. F. Cheng, X. X. Wang, H. Wu, Q. Ma, J. J. Liu, W. F. Feng, J. Nie, H. L. Yu, X. J. Huang, M. Armand, H. Zhang, Z. B. Zhou, *J. Power Sources* **2022**, 526, 231105; c) W. Wahyudi, X. Guo, V. Ladelta, L. Tsetseris, M. I. Nugraha, Y. Lin, V. Tung, N. Hadjichristidis, Q. Li, K. Xu, J. Ming, T. D. Anthopoulos, *Adv. Sci.* **2022**, 9, 2202405; d) L. Zheng, H. Zhang, P. Cheng, Q. Ma, J. Liu, J. Nie, W. Feng, Z. Zhou, *Electrochim. Acta* **2016**, 196, 169.

[12] H. Du, Y. Wang, Y. Kang, Y. Zhao, Y. Tian, X. Wang, Y. Tan, Z. Liang, J. Wozny, T. Li, D. Ren, L. Wang, X. He, P. Xiao, E. Mao, N. Tavajohi, F. Kang, B. Li, *Adv. Mater.* **2024**, 36, 2401482.

[13] a) B. Ravdel, K. M. Abraham, R. Gitzendanner, J. DiCarlo, B. Lucht, C. Campion. *J. Power Sources* **2003**, 119, 805; b) C. L. Campion, W. Li, B. L. Lucht. *J. Electrochem. Soc.* **2005** 152, A2327; c) B. Vortmann, S. Nowak, C. Engelhard. *Anal. Chem.* **2013**, 85, 3433; d) P. Handel, G. Fauler, K. Kapper, M. Schmuck, C. Stangl, R. Fischer, F. Uhlig, S. Koller. *J. Power Sources* **2014**, 267 255; e) W. Wahyudi, X. Guo, V. Ladelta, L. Tsetseris, M. I. Nugraha, Y. Lin, V. Tung, N. Hadjichristidis, Q. Li, K. Xu, J. Ming, T. D. Anthopoulos, *Adv. Sci.* **2022**, 9, 2202405; f) H. Du, Y. Wang, Y. Kang, Y. Zhao, Y. Tian, X. Wang, Y. Tan, Z. Liang, J. Wozny, T. Li, D. Ren, L. Wang, X. He, P. Xiao, E. Mao, N. Tavajohi, F. Kang, B. Li, *Adv. Mater.* **2024**, 36, 2401482.

[14] a) E. Wang, Y. Niu, Y.-X. Yin, Y.-G. Guo, *ACS Mater. Lett.* **2021**, 3, 18; b) G. G. Eshetu, S. Grugeon, G. Gachot, D. Mathiron, M. Armand, S. Laruelle, *Electrochim. Acta* **2013**, 102, 133.

[15] G. G. Eshetu, S. Grugeon, S. Laruelle, S. Boyanov, A. Lecocq, J.-P. Bertrand, G. Marlair, *Phys. Chem. Chem. Phys.* **2013**, 15, 9145.

[16] P. T. Bhutia, S. Grugeon, J.-P. Bertrand, G. Binotto, A. Bordes, A. El Mejdoubi, S. Laruelle, G. Marlair, *J. Power Sources* **2024**, 622, 235234.

[17] G. Bouteau, A. N. Van-Nhien, M. Sliwa, N. Sergent, J.-C. Lepretre, G. Gachot, I. Sagaidak, F. Sauvage, *Sci. Rep.* **2019**, 9, 135.

[18] G. G. Eshetu, H. Zhang, X. Judez, H. Adenusi, M. Armand, S. Passerini, E. Figgemeier, *Nat. Commun.* **2021**, 12, 5459.

[19] A. V. Cresce, S. M. Russell, O. Borodin, J. A. Allen, M. A. Schroeder, M. Dai, J. Peng, M. P. Gobet, S. G. Greenbaum, R. E. Rogers, K. Xu, *Phys. Chem. Chem. Phys.* **2017**, 19, 574.

[20] a) L. Haneke, J. E. Frerichs, A. Heckmann, M. M. Lerner, T. Akbay, T. Ishihara, M. R. Hansen, M. Winter, T. Placke, *J. Electrochem. Soc.* **2020**, 167, 140526; b) A. Ponrouch, R. Dedryvère, D. Monti, A. E. Demet, J. M. Ateba Mba, L. Croguennec, C. Masquelier, P. Johansson, M. R. Palacín, *Energy Environ. Sci.* **2013**, 6, 2361.

[21] Y.-S. Kim, S.-K. Jeong, *J. Spectro.* **2015**, 2015, 323649.

[22] R. Sakamoto, M. Yamashita, K. Nakamoto, Y. Zhou, N. Yoshimoto, K. Fujii, T. Yamaguchi, A. Kitajou, S. Okada, *Phys. Chem. Chem. Phys.* **2020**, 22, 26452.

[23] A. Kottarathil, Z. Slim, H. Ahmad Ishfaq, S. Jeschke, G. Z. Żukowska, M. Marczewski, K. Lech, P. Johansson, W. Wieczorek, *J. Electrochem. Soc.* **2024**, 171, 070506.

[24] The Materials Project. Materials Data on $LiAsF_6$ by Materials Project. United States, 2014.

[25] The Materials Project. Materials Data on $LiPF_6$ by Materials Project. United States, 2014.

[26] J. L. Nowinski, P. Lightfoot, P. G. Bruce, *J. Mater. Chem.* **1994**, 4, 1579.

[27] M. S. Wickleder, *Z. Anorg. Allg. Chem.* **2003**, 629, 1466.

[28] D. M. Seo, T. Afroz, J. L. Allen, P. D. Boyle, P. C. Trulove, H. C. De Long, W. A. Henderson, *J. Phys. Chem. C* **2014**, 118, 25884.

[29] R. Frech, C. P. Rhodes, M. Khan, *Macromol. Sympo.* **2002**, 186, 41.

[30] C. P. Rhodes, R. Frech, *Macromolecules* **2001**, 34, 2660.

[31] W. A. Henderson, F. McKenna, M. A. Khan, N. R. Brooks, V. G. Young, Jr., R. Frech, *Chem. Mater.* **2005**, 17, 2284.

[32] W. A. Henderson, N. R. Brooks, W. W. Brennessel, V. G. Young, *Chem. Mater.* **2003**, 15, 4679.

[33] W. A. Henderson, N. R. Brooks, W. W. Brennessel, V. G. Young, *J. Phys. Chem. A* **2004**, 108, 225.

[34] G. S. MacGlashan, Y. G. Andreev, P. G. Bruce, *Nature* **1999**, 398, 792.

[35] K. Kitashita, R. Hagiwara, Y. Ito, O. Tamada, *J. Fluorine Chem.* **2000**, 101, 173.

[36] L. Xue, C. W. Padgett, D. D. DesMarteau, W. T. Pennington, *Solid State Sci.* **2002**, 4, 1535.

[37] R. Wartchow, H. Berthold, *Z. Krist.* **1978**, 147, 307.

[38] Y. G. Andreev, G. S. MacGlashan, P. G. Bruce, *Phys. Rev. B* **1997**, 55, 12011.

[39] P. Lightfoot, M. A. Mehta, P. G. Bruce, *J. Mater. Chem.* **1992**, 2, 379.

[40] T. Mandai, R. Nozawa, S. Tsuzuki, K. Yoshida, K. Ueno, K. Dokko, M. Watanabe, *J. Phys. Chem. B* **2013**, 117, 15072.
